# Functional substitutions of amino acids that differ between GDF11 and GDF8 impact skeletal development and skeletal muscle

**DOI:** 10.26508/lsa.202201662

**Published:** 2023-01-11

**Authors:** John Lian, Ryan G Walker, Andrea D’Amico, Ana Vujic, Melanie J Mills, Kathleen A Messemer, Kourtney R Mendello, Jill M Goldstein, Krystynne A Leacock, Soraya Epp, Emma V Stimpfl, Thomas B Thompson, Amy J Wagers, Richard T Lee

**Affiliations:** 1 Department of Stem Cell and Regenerative Biology and the Harvard Stem Cell Institute, Harvard University, Cambridge, MA, USA; 2 Department of Molecular Genetics, Biochemistry and Microbiology, University of Cincinnati, Cincinnati, OH, USA; 3 Joslin Diabetes Center, Boston, MA, USA; 4 Paul F. Glenn Center for the Biology of Aging, Harvard Medical School, Boston, MA, USA

## Abstract

Growth differentiation factor 11 (GDF11) and GDF8 (MSTN) are closely related TGF-β family proteins that interact with nearly identical signaling receptors and antagonists. However, GDF11 appears to activate SMAD2/3 more potently than GDF8 in vitro and in vivo. The ligands possess divergent structural properties, whereby substituting unique GDF11 amino acids into GDF8 enhanced the activity of the resulting chimeric GDF8. We investigated potentially distinct endogenous activities of GDF11 and GDF8 in vivo by genetically modifying their mature signaling domains. Full recoding of GDF8 to that of GDF11 yielded mice lacking GDF8, with GDF11 levels ∼50-fold higher than normal, and exhibiting modestly decreased muscle mass, with no apparent negative impacts on health or survival. Substitution of two specific amino acids in the fingertip region of GDF11 with the corresponding GDF8 residues resulted in prenatal axial skeletal transformations, consistent with *Gdf11*-deficient mice, without apparent perturbation of skeletal or cardiac muscle development or homeostasis. These experiments uncover distinctive features between the GDF11 and GDF8 mature domains in vivo and identify a specific requirement for GDF11 in early-stage skeletal development.

## Abstract (precis)

Replacement of amino acids unique to GDF11 and GDF8 into the native locus of the other ligand yields measurable, differential skeletal and muscle phenotypes, revealing distinct features between the ligands and a requirement for GDF11 in early-stage skeletal development.

## Introduction

**Figure 1.**
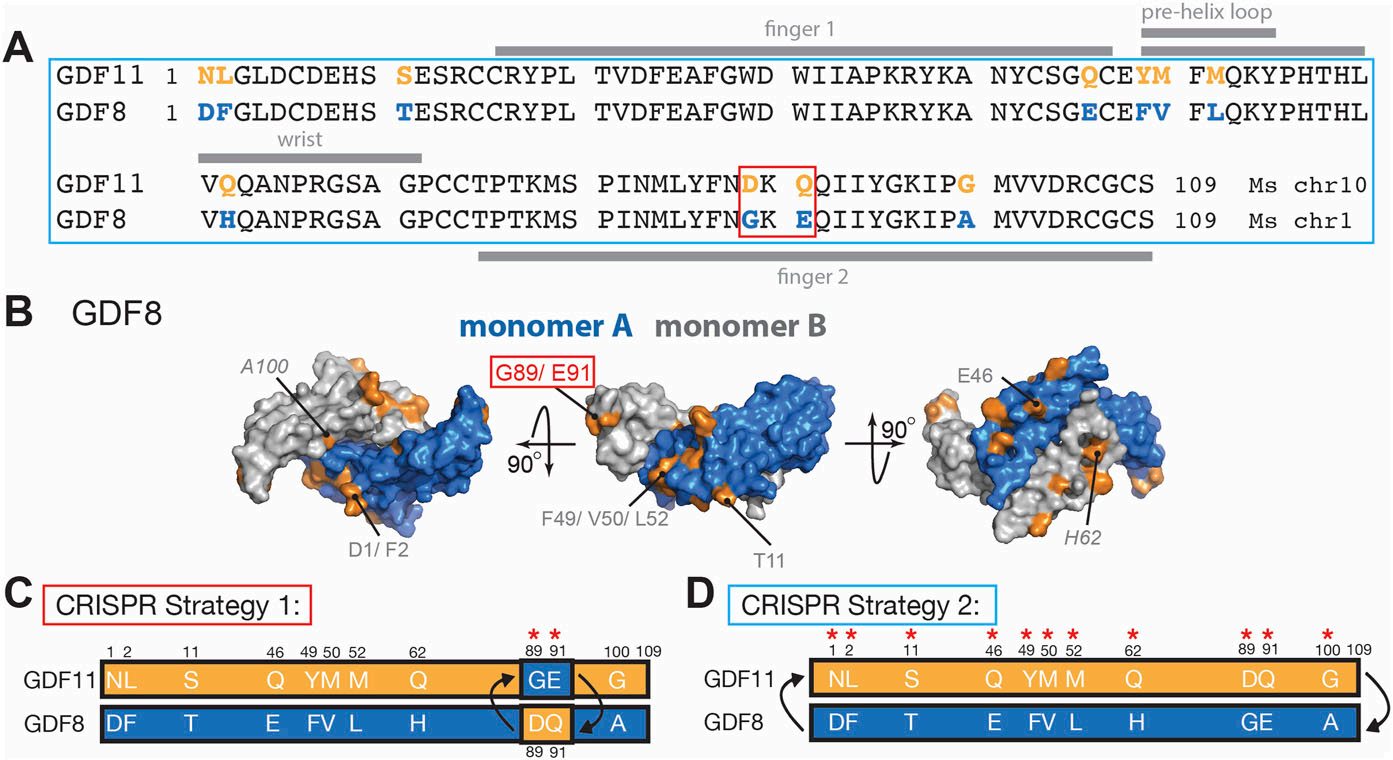
Sequence differences between *Gdf11* and *Gdf8* and CRISPR/Cas9 strategy to substitute amino acid residues within GDF11 and GDF8 mature domains. **(A)** *Gdf11* unique amino acid residues are highlighted in orange and *Gdf8* unique residues in blue. **(B)** Surface representation of GDF8 (monomer A in blue and monomer B in grey) showing location of unique GDF8 amino acid residues in orange, with G89 and E91 highlighted in red. **(C)** Schematic of CRISPR/Cas9 strategies for changing residues in *Gdf11* and *Gdf8* native loci. In Strategy 1, the D89 and Q91 amino acid residues within *Gdf11* are changed for the analogous G89 and E91 amino acid residues from *Gdf8*, and the G89 and E91 amino acid residues within *Gdf8* are changed for the analogous D89 and Q91 residues from *Gdf11*. **(D)** In Strategy 2, the full mature domain of *Gdf8* is changed for the full mature domain from *Gdf11*. Amino acid residues of *Gdf11* are shown in orange, and those of *Gdf8*, in blue.

The TGF-β superfamily of proteins is well known for regulating embryological development, wound healing, and adult tissue maintenance. In recent years, two highly homologous TGF-β proteins—growth differentiation factor 11 (GDF11) and GDF8 (also known as myostatin/MSTN)—have garnered substantial interest with evidence of their roles in aging and regenerative processes (Loffredo et al, 2013; Biesemann et al, 2014; Sinha et al, 2014; Poggioli et al, 2016; Walker et al, 2016; Du et al, 2017). Due to the 89% amino acid sequence identity in their C-terminal signaling domains, GDF11 and GDF8 have been viewed as serving redundant functions in vivo (McPherron et al, 2009; Poggioli et al, 2016; Walker et al, 2016) (Fig 1A). Yet, growing evidence suggests that GDF11 and GDF8 have distinct potencies and different spatiotemporal functions in vivo. As members of the activin subclass of TGF-β, both GDF8 and GDF11 signal through the type I receptors ALK4, ALK5, and ALK7 (Rebbapragada et al, 2003; Andersson et al, 2006; Walker et al, 2017; Lee et al, 2020). Molecularly, they are synthesized as precursors that remain in an inactive, latent complex until a Tolloid-like (TLD) protease cleaves the ligand prodomain to relieve the mature domain from inhibition (Lee & McPherron, 2001; Rebbapragada et al, 2003; Wolfman et al, 2003; Ge et al, 2005; McFarlane et al, 2005; Anderson et al, 2008). Mature GDF11 and mature GDF8 each consist of two monomers linked by disulfide bonds to form a homodimer of propeller-like shape (Fig 1B), which creates symmetrical concave and convex surfaces used for receptor binding (Yadin et al, 2016; Walker et al, 2017). To signal, the ligands assemble a combination of two type II and two type I Ser/Thr kinase receptors that have a single extracellular ligand-binding domain (Allendorph et al, 2006; Weber et al, 2007). Assembly of this complex allows the type II receptor to phosphorylate the type I receptor, which initiates the SMAD signaling cascade (Weiss & Attisano, 2013).

GDF8 is expressed postnatally, predominantly by skeletal and cardiac muscles, and is a well-recognized negative regulator of muscle growth (McPherron & Lee, 1997; McPherron et al, 1997; Lee, 2012). GDF11 is expressed more broadly across multiple tissues, with noted involvement in early development (McPherron et al, 1999; Wu et al, 2003; Kim et al, 2005; Liu, 2006; McPherron et al, 2009). Genetic mutation of *Mstn* (*Gdf8*) results in hypermuscular, hypo-adipose phenotypes in numerous animal species, including humans (McPherron & Lee, 1997; McPherron et al, 1997; Schuelke et al, 2004; Clop et al, 2006; Mosher et al, 2007), whereas homozygous deletion of *Gdf11* leads to axial skeletal malformation and defects in organ development in mice (McPherron et al, 1999). In addition, recent evidence suggests that genetic loss of GDF11 function in humans causes multisystem pathology with variable impact on the skeleton, nervous system, heart, muscle, and/or connective tissue (Ravenscroft et al, 2021). Importantly, *Gdf11*-null mice exhibit perinatal lethality, whereas *Mstn*-null (*Gdf8*^*−/−*^) mice do not (McPherron et al, 1999), and lower levels of *Mstn* in *Gdf8*^*+/−*^ heterozygotes may actually extend lifespan (Mendias et al, 2015). These differences in postnatal survival following genetic manipulation make comparative studies of *Gdf11* versus *Gdf8* activities in vivo particularly difficult, while reports on GDF11’s essential functions in adulthood—most of which have relied on the use of exogenous recombinant proteins—are incompletely defined. Nevertheless, interest persists from pharmaceutical and biotechnology companies in the potential effects of GDF11 in age-related organ dysfunction (Loffredo et al, 2013; Katsimpardi et al, 2014; Sinha et al, 2014), and several studies support the notion that exogenous GDF11 may regulate cardiac hypertrophy and skeletal muscle repair in older animals (Loffredo et al, 2013; Sinha et al, 2014; Du et al, 2017).

In a prior study, we demonstrated that GDF11 and GDF8 differ in their signaling properties in multiple cell lines and cultured primary myoblasts, with GDF11 signaling at lower concentrations than GDF8 and more efficiently using the type I receptors ALK4, ALK5, and ALK7 (Walker et al, 2017). We define this ability to activate downstream pathways at lower concentrations as having greater *potency*. We further showed that administration of GDF11 in vivo more potently induces SMAD phosphorylation in the myocardium compared with GDF8 (Walker et al, 2017). These differences implicate residue differences between GDF11 and GDF8, particularly those clustered around the type I binding interface, in determining signaling potency, likely via effects on dimer stability and stability of receptor interactions. Consistent with this possibility, structural analysis and mutational studies of the ternary complex of GDF11 with type I receptor Alk5 and type II receptor ActRIIB revealed that different mechanisms regulate specificity and binding with type I receptor, compared with TGF-β, providing an explanation for how GDF11 and the TGF-β activin class more effectively facilitate low-affinity type I interactions (Goebel et al, 2019). These biochemical and structural studies indicate that GDF11 and GDF8 are unlikely to be functionally equivalent, especially when ligand concentrations are low, as they typically are in vivo (Walker et al, 2017; Goebel et al, 2019).

In this study, we evaluate GDF11 and GDF8 functional equivalence in vivo by using the CRISPR/Cas9 system to introduce GDF8-like amino acid substitutions into GDF11, and GDF11-like substitutions into GDF8. These sequence alterations in GDF11, which previously were shown to diminish signaling potency of the resulting protein (Walker et al, 2017), caused a perturbation of the axial skeletal structure of mutant mice during development that persists into adulthood. In contrast, the sequence alterations introduced into GDF8, which previously were shown to increase the signaling potency of the resulting ligand (Walker et al, 2017), did not produce observable developmental phenotypes. As such, we generated a third line of mutant animals, in which the entire GDF8 mature domain was replaced with the corresponding mature domain sequence of GDF11, resulting in full replacement of the endogenous GDF8 signaling domain with that of GDF11. These mature domain (MD) mutants had up to 50-fold greater levels of GDF11 in circulation, with concomitant depletion of GDF8 to undetectable levels and showed modestly decreased skeletal muscle mass, with no apparent impact on postnatal survival, total adult body weight, or the development and function of other organ systems.

While we were performing our study, the Se-jin Lee group published a study that used a similar genetic approach as our third mouse line to replace the *Mstn* gene sequences encoding the mature C-terminal peptide with the full mature domain of *Gdf11* (Lee et al, 2022). Their characterization of these mice (Lee et al, 2022) supports our data here, showing that GDF11 entirely replaced circulating MSTN and increased GDF11 levels ∼30–40-fold (Lee et al, 2022). However, our findings extend these observations, addressing the converse hypothesis as well and showing that diminution of GDF11 potency—through targeted replacement of two key amino acids from GDF8—causes a significant developmental defect in osteogenesis, distinct from that seen with modulation of GDF8. Taken together, our findings elucidate precise, differential molecular mechanisms underpinning the biological actions of GDF11 and GDF8 that cannot be explained solely by differences in in vivo ligand concentrations and patterns of expression. They also provide direct evidence that structural and biochemical differences in these ligand’s mature signaling domains contribute significantly to their unique roles in mammalian development and organ physiology.

## Results

### Generation and characterization of chimeric amino acid GDF11 and GDF8 mice

We previously reported that substitution of two residues located in the fingertip of GDF11 (D89 and Q91) into the analogous region of GDF8 (in place of G89 and E91) enhanced SMAD signaling activity of the hybrid GDF8 molecule by ∼50% (Walker et al, 2017). This result indicates that sequence differences in the mature GDF11 and GDF8 proteins are likely responsible for differences in ligand signaling and function. To address whether such sequence-determined signaling differences impact in vivo activities of GDF11 and GDF8, we used CRISPR/Cas9 to create two lines of chimeric mice (Strategy 1; Fig 1C), in which we replaced D89 and Q91 residues within the *Gdf11* locus with the analogous G89 and E91 residues from *Gdf8*, or conversely replaced G89 and E91 within native *Gdf8* with D89 and Q91 from *Gdf11*. The third chimeric line we generated replaced the full mature domain region within the *Gdf8* locus with the corresponding region from *Gdf8* (Strategy 2; Fig 1D). Based on our prior in vitro studies, substitution of all the unique GDF11 residues into GDF8 in this manner is able to enhance signaling of the resulting protein to be ∼5-fold more potent than WT GDF8 (Walker et al, 2017).

**Figure S1.**
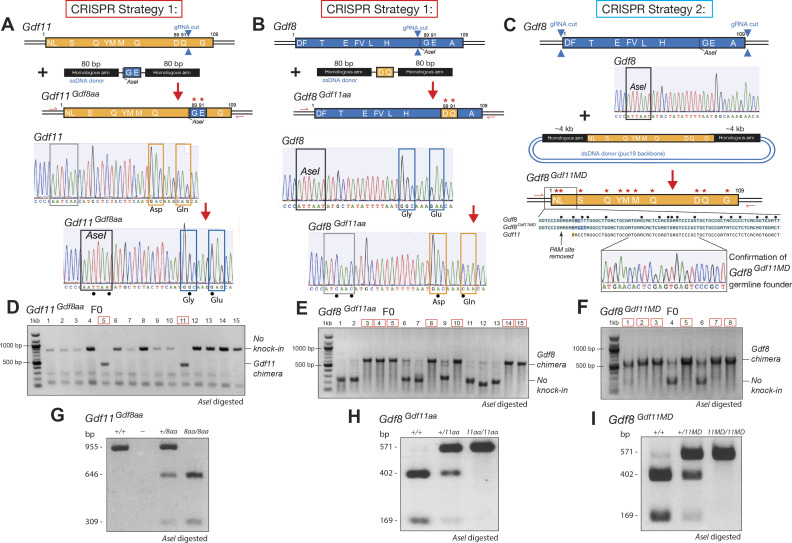
CRISPR/Cas9 approach and characterization for generating GDF11 and GDF8 chimeric mice. To generate *Gdf11*^*Gdf8aa*^, *Gdf8*^*Gdf11aa*^, and *Gdf8*^*Gdf11MD*^ mutant lines, DNA donor plasmids were designed to contain the desired amino acid or full mature domain mutations. **(A, B, C)** For the amino acid mutant lines (A) *Gdf11*^*Gdf8aa*^ and (B) *Gdf8*^*Gdf11aa*^, single-stranded donor templates were flanked by 80-bp homologous arms, whereas a double-stranded donor template was flanked by 4-kb homologous arms, which was constructed for the full mature domain mutant (C) *Gdf8*^*Gdf11MD*^. Schematics of the CRISPR strategy depict the native *Gdf11* and *Gdf8* loci, with locations of the gRNA cut site (top), the donor templates containing the codons to incorporate the *Gdf8*-like and *Gdf11*-like amino acid mutations (middle), and the resulting chimeric mutants (bottom). **(A, B, C, D, E, F)** Successful transgene modification was confirmed via Sanger sequencing (A, B, C) and PCR validation and subcloning, identifying F0 founders for (D) *Gdf11*^*Gdf8aa*^, (E) *Gdf8*^*Gdf11aa*^, and (F) *Gdf8*^*Gdf11MD*^ mouse lines. **(G)** In the process of genotyping, digested bands from incubation of DNA with restriction enzyme *AseI* were shown for *Gdf11*^*+/8aa*^ and *Gdf11*^*8aa/8aa*^ samples, which acquired the *AseI* site from the donor template. **(H)** *Gdf8*^*+/11aa*^ and *Gdf8*^*11aa/11aa*^ DNA could no longer be digested fully after incubation with *AseI*, indicating the absence of the excised native *AseI* site. **(I)** *Gdf8*^*+/11MD*^ and *Gdf8*^*11MD/11MD*^ DNA also could not be digested fully after incubation with *AseI*. Amino acid residues of *Gdf11* are represented in orange, and those of *Gdf8*, in blue.

To generate the two amino acid–modified lines, we constructed genetically modified *Gdf11* (Fig S1A) and *Gdf8* (Fig S1B) single-stranded DNA (ssDNA) donor plasmids containing the mutant codons, flanked by ∼80-bp homologous arms. In generating the full mature domain replacement line, we constructed a genetically modified *Gdf8* double-stranded DNA (dsDNA) donor plasmid (Fig S1C) containing the GDF11 mature domain sequences, flanked by ∼4-kb homologous arms. After homologous recombination in embryonic stem cells, targeted microinjections into C57BL/6J zygotes, and implantation of zygotes into C57BL/6J surrogate females, we produced F0 founders with the chimeric allele incorporated in the germline. The ssDNA donor template incorporating *Gdf8*-like G89 and E91 residues into the native *Gdf11* locus (Fig S1A) also introduced an *AseI* restriction enzyme unique to *Gdf8* as a genetic marker for downstream genotyping. Likewise, the ssDNA donor template incorporating *Gdf11*-like D89 and Q91 residues into the native *Gdf8* locus (Fig S1B) and the dsDNA donor template containing the *Gdf11* full mature domain sequences (Fig S1C) removed the same *AseI* site from the *Gdf8* locus. We verified successful integration of the chimeric constructs at the *Gdf11* and *Gdf8* loci via Sanger sequencing (Fig S1A–C) and identified F0 founders through PCR validation and subcloning for *Gdf11*^*Gdf8aa*^ (Fig S1D), *Gdf8*^*Gdf11aa*^ (Fig S1E), and *Gdf8*^*Gdf11MD*^ (Fig S1F) lines. Downstream genotyping confirmed the presence of the unique *AseI* restriction enzyme site in the modified *Gdf11* locus (Fig S1G) and the absence of the same *AseI* site at the modified *Gdf8* loci (Fig S1H and I). We further confirmed integration of silent mutations included in the donor templates, whose purpose was to mutate the PAM sequence to prevent further cutting after donor construct integration (Fig S1A–C). Collectively, these results validated our genetic modification strategy to integrate *Gdf11*-like and *Gdf8*-like changes into the *Gdf8* and *Gdf11* loci, respectively.

Through this process, we successfully generated the following:

(1) *Gdf11*^*Gdf8aa*^ mice (with *Gdf8* amino acid residues G89 and E91 replacing the corresponding residues in *Gdf11*) (Fig S1A).
  (a) Mono-allelic (*Gdf11*^*+/8aa*^), bi-allelic (*Gdf11*^*8aa/8aa*^), WT (*Gdf11*^*+/+*^).
(2) *Gdf8^Gdf11aa^* mice (with *Gdf11* amino acid residues D89 and Q91 replacing the corresponding residues in *Gdf8*) (Fig S1B).
  (a) Mono-allelic (*Gdf8^+/11aa^*), bi-allelic (*Gdf8^11aa/11aa^*), WT (*Gdf8^+/+^*).
(3) *Gdf8^Gdf11MD^* mice (with the Gdf11 mature domain replacing the Gdf8 mature domain in the Gdf8 locus) (Fig S1C).
  (a) Mono-allelic (*Gdf8^+/11MD^*), bi-allelic (*Gdf8^11MD/11MD^*), WT (*Gdf8^+/+^*).

**Figure S2.**
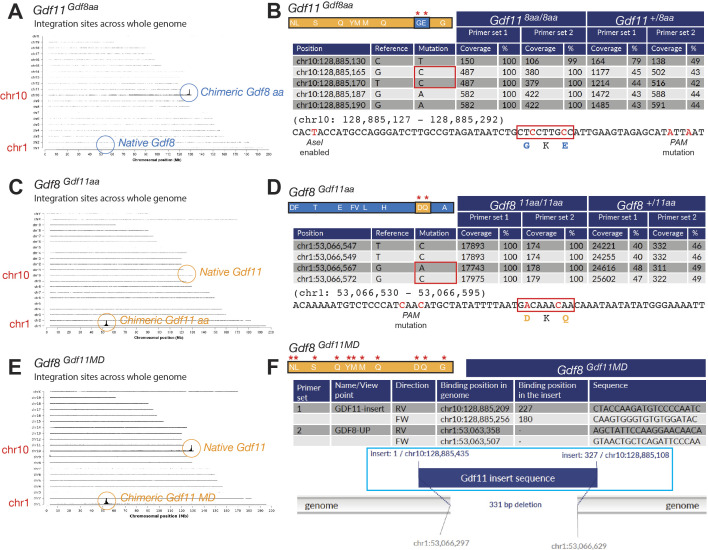
Genotyping and targeted locus amplification sequencing of whole-genome integration events in *Gdf11*^*Gdf8aa*^, *Gdf8*^*Gdf11aa*^, and *Gdf8*^*Gdf11MD*^ mutants. **(A)** In *Gdf11*^*Gdf8aa*^ mice, detected mutations were in exon 3 of *Gdf11* on chr10 (RefSeq). **(B)** In *Gdf11*^*8aa/8aa*^ samples, two mutated bases (G→C = D89G and T→C = Q91E) were found at 100% frequency, indicating bi-allelic mutation. In *Gdf11*^*+/8aa*^ samples, the mutations occurred at ∼50% frequency, indicating mono-allelic mutation. Three additional silent mutations—one for the insertion of the *AseI* site unique to *Gdf8* and two for the mutations—were also confirmed. **(C)** In *Gdf8*^*Gdf11aa*^ mice, mutations were in exon 3 of *Gdf8* on chr1 (RefSeq). **(D)** In *Gdf8*^*11aa/11aa*^ samples, two mutated bases (G→A = G89D and G→C = E91Q) were found at 100% frequency, indicating bi-allelic mutation. In *Gdf8*^*+/11aa*^ samples, the mutations occurred at ∼50% frequency, indicating mono-allelic mutation. Two additional base changes, accounting for the PAM site silent mutation, and the absence of the *AseI* site were also confirmed. **(E)** In *Gdf8*^*Gdf11MD*^ mutants, the full GDF11 mature domain sequence was found on chr1, in addition to the native *Gdf8* locus on chr10. No structural variants, off-target integration, or locus duplication was detected. **(F)** *Gdf8*^*11MD/11MD*^ samples lacked WT *Gdf8* mature domain sequences at the integration site and in the deleted region, confirming bi-allelic replacement of native GDF8, whereas *Gdf8*^*+/11MD*^ samples had WT reads at the integration site and in the deleted region on one allele, confirming mono-allelic replacement of native GDF8. The mouse mm10 reference sequence was used for comparisons. Amino acid residues of *Gdf11* are shown in orange, and those of *Gdf8*, in blue.

We backcrossed the knock-in alleles five generations (to F5) in each chimeric line before analyses to breed out potential off-target modifications and confirmed Mendelian ratios of allele inheritance to rule out potential embryonic lethality resulting from the genetic modifications (Table S1). Targeted locus amplification (TLA) sequencing (de Vree et al, 2014), performed on bone marrow DNA harvested from F5 mono-allelic and bi-allelic offspring from *Gdf11*^*Gdf8aa*^ (Fig S2A and B), *Gdf8*^*Gdf11aa*^ (Fig S2C and D), and *Gdf8*^*Gdf11MD*^ (Fig S2E and F) mice and aligned to the mouse mm10 genome sequence, further confirmed correct integration of the desired mutant sequences into the native *Gdf11* and *Gdf8* loci, with no evidence across the whole genome of structural variation surrounding the integration site or within the insert, of incorrect or off-target integration events, or of a locus duplication at the integration site matching the WT allele. Taken together, these results indicate that the *Gdf11*^*Gdf8aa*^ and *Gdf8*^*Gdf11aa*^ chimeric lines successfully integrated the intended *Gdf8* or *Gdf11* nucleotide alterations leading to the anticipated amino acid changes, with no other genomic off-target mutations detected, and that the *Gdf8*^*Gdf11MD*^ line successfully integrated the full mature domain sequences of *Gdf11* and replaced the native mature domain region of *Gdf8*, with no evidence of incorrect targeting.

Table S1 Genotype and survival distribution of *Gdf11*^*Gdf8aa*^, *Gdf8*^*Gdf11aa*^, and *Gdf8*^*Gdf11MD*^ F5 progeny in C57BL/6J background through three initial rounds of breeding.

### Circulating GDF11 concentration increases 50-fold in *Gdf8*^*Gdf11MD*^ mutants, whereas GDF11 and GDF8 levels in *Gdf11*^*Gdf8aa*^ and *Gdf8*^*Gdf11aa*^ mutants remain unchanged

**Figure 2.**
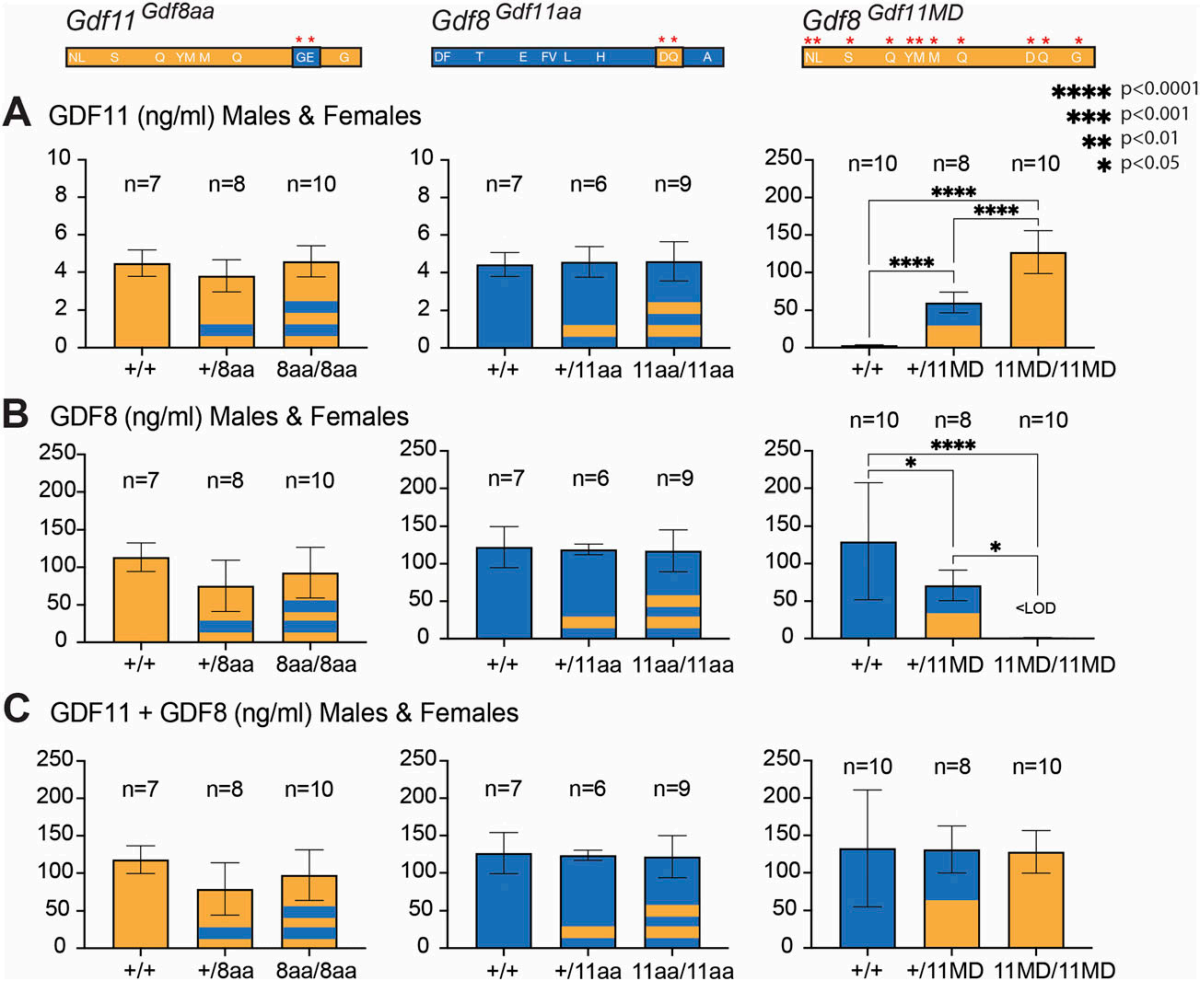
GDF11 and GDF8 serum concentrations in *Gdf11*^*Gdf8aa*^, *Gdf8*^*Gdf11aa*^, and *Gdf8*^*Gdf11MD*^ mice. **(A, B)** Liquid chromatography–tandem mass spectrometry measurements of (A) GDF11 and (B) GDF8 in *Gdf11*^*+/+*^ (n = 7), *Gdf11*^*+/8aa*^ (n = 8), and *Gdf11*^*8aa/8aa*^ (n = 10) mice (left plots); in *Gdf8*^*+/+*^ (n = 7), *Gdf8*^*+/11aa*^ (n = 6), and *Gdf8*^*11aa/11aa*^ (n = 9) mice (middle plots); and in *Gdf8*^*+/+*^ (n = 10), *Gdf8*^*+/11MD*^ (n = 8), and *Gdf8*^*11MD/11MD*^ (n = 10) mice (right plots). <LOD, below the level of detection. The same trends were observed in male and female mice separately. **(C)** Combined concentration of GDF11 and GDF8 in *Gdf11*^*Gdf8aa*^, *Gdf8*^*Gdf11aa*^, and *Gdf8*^*Gdf11MD*^ mice as a measure of total ligand levels. Statistical analyses were performed by one-way ANOVA with Tukey’s correction for multiple comparisons. Amino acid residues of *Gdf11* are represented in orange, and those of *Gdf8*, in blue. For *Gdf11*^*Gdf8aa*^ and *Gdf8*^*Gdf11aa*^ lines, one stripe denotes mono-allelic replacement, and two stripes denote bi-allelic replacement. For *Gdf8*^*Gdf11MD*^ mice, half orange denotes mono-allelic replacement, and full orange denotes bi-allelic replacement.

To determine whether full replacement of the mature domain of native *Gdf8* with *Gdf11* altered circulating protein levels in vivo, we collected serum from *Gdf8*^*+/+*^, *Gdf8*^*+/11MD*^, and *Gdf8*^*11MD/11MD*^ mice at 10–14 wk of age and measured endogenous GDF11 and GDF8 levels, using a liquid chromatography–tandem mass spectrometry assay that distinguishes between GDF11 and GDF8 by detecting two differential peptide fragments between the two proteins (Garbern et al, 2019) (Fig 2). In the bi-allelic *Gdf8*^*11MD/11MD*^ mutants (n = 10), circulating GDF11 concentrations increased ∼50-fold above normal levels (Fig 2A), whereas circulating GDF8 concentrations decreased below the level of detection (<LOD) (Fig 2B). The same trend in GDF11 and GDF8 concentrations was observed in male and female mice separately, with significant differences in ligand concentration varying according to allelic dosage across the three genotypes (*Gdf8*^*+/+*^, n = 10; *Gdf8*^*+/11MD*^, n = 8; and *Gdf8*^*11MD/11MD*^, n = 10). The levels of GDF11 peptides measured in *Gdf8*^*11MD/11MD*^ mutants (Fig 2A) were comparable to the levels of GDF8 in *Gdf8*^*+/+*^ mice (Fig 2B) and to the combined concentrations of GDF11 + GDF8 in *Gdf8*^*+/11MD*^ mice (Fig 2A and B), suggesting a direct replacement of mature GDF11 for GDF8, with expression levels ultimately determined by either cis-regulatory elements in the *Gdf8* locus or association with the GDF8 prodomain, or a combination of these two factors. In support of this interpretation, the total pool of GDF11 + GDF8 did not differ for any of the chimeric genotypes (Fig 2C). Mass spectrometry data from the Se-jin Lee group of the *Gdf8* full coding region–replaced chimeras corroborate our findings (Lee et al, 2022). They also reported no detectable MSTN and a ∼30–40-fold increase in circulating GDF11 in *Mstn*^*Gdf11/Gdf11*^ mice (Lee et al, 2022), with *Mstn*^*+/Gdf11*^ mice having intermediate levels of the two ligands (Lee et al, 2022). Our study further expands this analysis of the impact of mature domain sequence on ligand expression by measuring endogenous GDF11 and GDF8 in serum from *Gdf11*^*Gdf8aa*^ and *Gdf8*^*Gdf11aa*^ mutants as well, showing that serum GDF11 and GDF8 protein concentrations are not significantly altered in either mono-allelic or bi-allelic *Gdf11*^*Gdf8aa*^ or *Gdf8*^*Gdf11aa*^ mutants, compared with WT mice (Fig 2A–C). Based on the biochemistry of the ligands, we do not anticipate that the matrix binding of GDF11 and GDF8 would be affected, though we have not studied this. These data indicate that the dual amino acid substitutions alone did not impact endogenous expression or circulation of GDF11 or GDF8. Therefore, despite some reports that high levels of GDF11 in humans are associated with adverse health consequences (Egerman et al, 2015: Hinken et al, 2016; Hammers et al, 2017), our data, together with those of Lee and colleagues (Lee et al, 2022), indicate that GDF11 can rise to extremely high levels in vivo without apparent negative health consequences or premature death.

### GDF11 dampening in bi-allelic *Gdf11*^*Gdf8aa*^ mutant embryos recapitulates developmental phenotype seen with *Gdf11* loss of function

**Figure 3.**
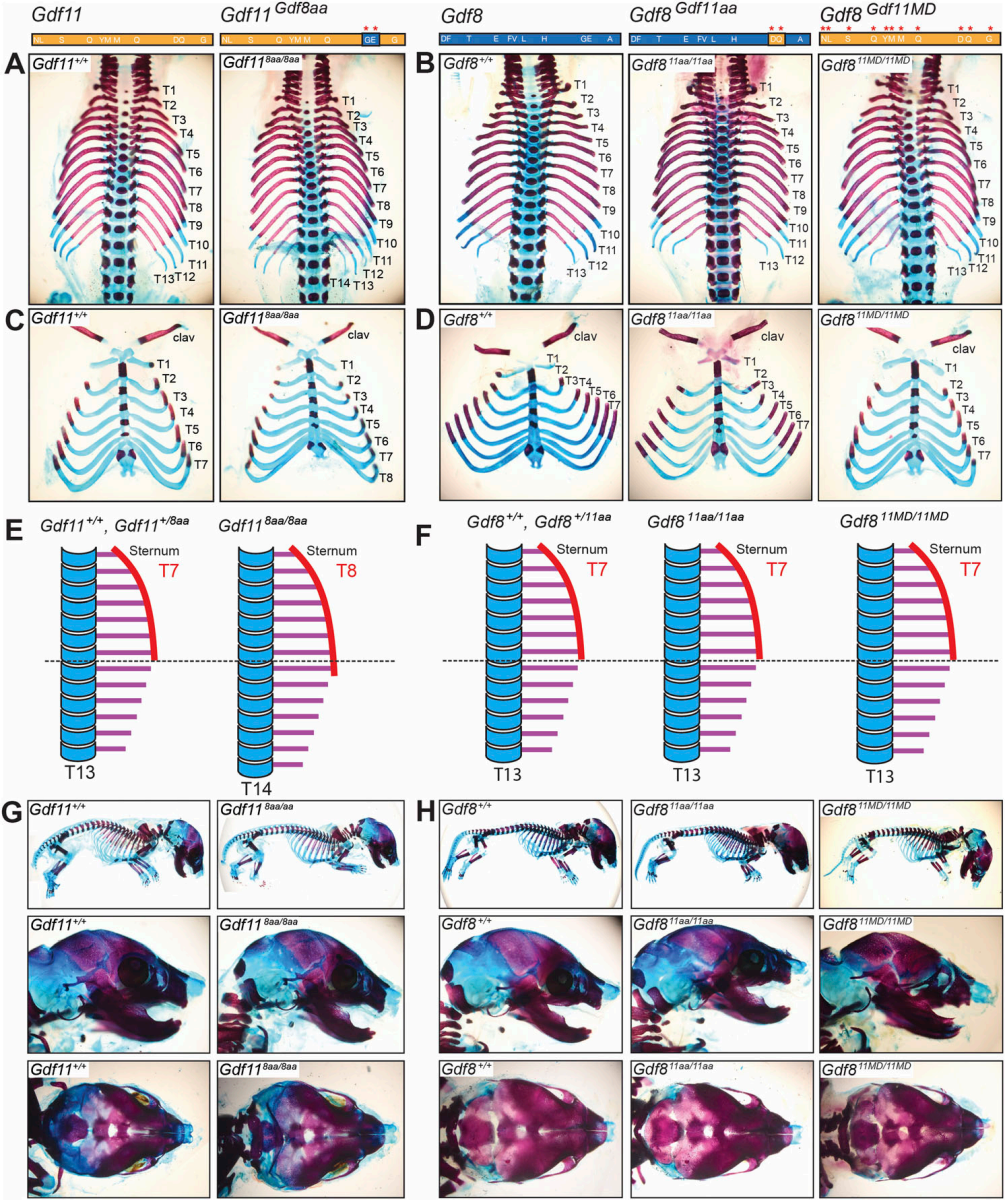
Axial skeletal patterning, craniofacial bone, and limb development in *Gdf11*^*Gdf8aa*^, *Gdf8*^*Gdf11aa*^, and *Gdf8*^*Gdf11MD*^ mice. Skeletons from *Gdf11*^*Gdf8aa*^, *Gdf8*^*Gdf11aa*^, and *Gdf8*^*Gdf11MD*^ embryos were harvested at E18.5 and stained with Alizarin red and Alcian blue. *Gdf11*^*+/+*^ (leftmost), *Gdf11*^*8aa/8aa*^ (second from left), *Gdf8*^*+/+*^ (middle), *Gdf8*^*+/11aa*^ (second from right), and *Gdf8*^*11MD/11MD*^ (rightmost) vertebral columns, vertebrosternal ribs, full skeletons, and craniofacial bones are shown. **(A)** *Gdf11*^*8aa/8aa*^ skeletons exhibited abnormal axial vertebral patterning with T14 total axial vertebrae, compared with T13 total vertebrae in *Gdf11*^*+/+*^ skeletons. **(B)** All other mutant skeletons also had T13 total vertebrae. **(C, D)** *Gdf11*^*8aa/8aa*^ skeletons also had T8 vertebrosternal ribs, compared with T7 vertebrosternal ribs in *Gdf11*^*+/+*^ skeletons and (D) in other mutant skeletons. **(E, F)** Schematic of axial vertebral columns in (E) *Gdf11*^*Gdf8aa*^ embryos and in (F) *Gdf8*^*Gdf11aa*^ and *Gdf8*^*Gdf11MD*^ embryos. Mono-allelic mutant mice *Gdf11*^*+/8aa*^, *Gdf8*^*+/11aa*^, and *Gdf8*^*+/11MD*^ also had T13 total axial vertebrae and T7 vertebrosternal ribs. **(G, H)** In *Gdf11*^*8aa/8aa*^ and (H) *Gdf8*^*11aa/11aa*^ and *Gdf8*^*11MD/11MD*^ mutants, the full skeleton and craniofacial bones, in profile (middle row) and from above (bottom row), were indistinguishable from *Gdf11*^*+/+*^ and *Gdf8*^*+/+*^ mice, respectively. The limbs and digits were also similar to WT. Amino acid residues of *Gdf11* are shown in orange, and those of *Gdf8*, in blue.

Germline deletion of *Gdf11* results in perinatal lethality, and both homozygous (*Gdf11*^*−/−*^) and heterozygous (*Gdf11*^*+/−*^) disruption of *Gdf11* cause developmental abnormalities of the skeleton (McPherron et al, 1999; Walker et al, 2016)—notably, the formation of extrathoracic vertebrae—and kidney agenesis in pups (Esquela & Lee, 2003; McPherron et al, 2009). We therefore assessed early-stage skeletal development in our chimeric embryos (Fig 3). Six sets of F4 mono-allelic mutant males were bred with mono-allelic mutant females within each *Gdf11*^*Gdf8aa*^, *Gdf8*^*Gdf11aa*^, and *Gdf8*^*Gdf11MD*^ mouse line to generate F5 embryos that were harvested on embryonic day 18.5 (E18.5), eviscerated, and stained with Alizarin red and Alcian blue (Fig 3). Before evisceration, tissue samples from the posterior skin of prenatal pups were collected for genotyping by PCR validation and subcloning. Spleen and liver samples were also taken from each embryo for genotyping (data not shown) to confirm that maternal DNA from the fallopian tubes and gestational sacs would not obfuscate genotyping results by contaminating the collected embryonic tissue.

Upon imaging the skeletons, we discovered distinct skeletal transformations in the *Gdf11*^*Gdf8aa*^ mouse line. Specifically, the bi-allelic *Gdf11*^*8aa/8aa*^ mutants exhibited one extrathoracic vertebra, with T14 axial vertebrae in total, compared with T13 vertebrae in WT mice (Fig 3A and E). We also observed an extra vertebrosternal rib in *Gdf11*^*8aa/8aa*^ mutants, resulting in a total of T8 ribs connected to the sternum, compared with T7 ribs in *Gdf11*^*+/+*^ and *Gdf11*^*+/8aa*^ mice (Fig 3C and E). Phenotypically, this differential development directly compares to the axial skeletal phenotype observed in heterozygous *Gdf11*^*+/−*^ mice, which also have 14 total thoracic vertebrae and 8 pairs of ribs fused to the sternum (McPherron et al, 1999). In contrast, homozygous *Gdf11*^*−/−*^ knockout mice have 17–18 total thoracic vertebrae and 10–11 pairs of vertebrosternal ribs (McPherron et al, 1999). We further verified that the malformation documented occurred only in *Gdf11*^*8aa/8aa*^ mutants, and with 100% penetrance (Table 1). Moreover, given that circulating ligand levels in these chimeric mice remained unaltered, the observed differential skeletal phenotypes are specifically attributable to the dual amino acid changes made in the protein sequences and structures—not to alterations of circulating protein levels. In contrast, the *Gdf8*^*Gdf11aa*^ mutant skeletons appeared indistinguishable from WT (Fig 3H), and none of the mono-allelic or bi-allelic mutants produced a measurable skeletal phenotype (Fig 3B, D, and F and Table 1). As stated previously, we generated the *Gdf8*^*Gdf11MD*^ mutant line to investigate whether increasing GDF8 potency to the maximum level of GDF11, by replacing the entire mature domain of GDF8 with that of GDF11, would result in observable phenotypic outcomes. However, axial skeletal analysis of *Gdf8*^*Gdf11MD*^ mice also revealed no measurable defects in the mutant skeletons, compared with WT (Fig 3B, D, and F and Table 1). Reported analyses of the related *Mstn*^*Gdf11/Gdf11*^ line generated by Lee and colleagues similarly found no abnormalities of axial skeletal patterning, though some decrement in bone density and alterations in trabeculae were noted, almost exclusively in males (Lee et al, 2022). These authors did not evaluate the impact of the dual amino acid substitutions reported here.

**Table 1.** Skeletal analysis of *Gdf11*^*Gdf8aa*^, *Gdf8*^*Gdf11aa*^, and *Gdf8*^*Gdf11MD*^ mouse embryos in C57BL/6J background.

Mouse line	*Gdf11*^*Gdf8aa*^ (n = 48)			*Gdf8Gdf11aa* (n = 53)			*Gdf8Gdf11MD* (n = 39)		
Genotype	+/+	+/8aa	8aa/8aa	+/+	+/11aa	11aa/11aa	+/+	+/11MD	11MD/11MD
n =	8	22	18	11	20	22	13	16	10
Vertebral pattern									
13th pair of ribs	8 (100)	22 (100)	—	11 (100)	20 (100)	22 (100)	13 (100)	16 (100)	10 (100)
14th pair of ribs	—	—	18 (100)	—	—	—			
Seven sternum ribs	8 (100)	22 (100)	—	11 (100)	20 (100)	22 (100)	13 (100)	16 (100)	10 (100)
Eight sternum ribs	—	—	18 (100)	—	—	—	—	—	—
T14 pair of ribs									
Intact pair	—	—	16 (88.9)	—	—	—	—	—	—
One side missing	—	—	2 (11.1)	—	—	—	—	—	—

Comparison of vertebral columns and vertebrosternal ribs from embryos harvested at E18.5. In *Gdf11*^*Gdf8aa*^ mice, T14 total axial vertebrae and T8 vertebrosternal ribs were observed in *Gdf11*^*8aa/8aa*^ embryos with 100% penetrance, compared with T13 vertebrae and T7 vertebrosternal ribs in 100% of *Gdf11*^*+/+*^ and *Gdf11*^*+/8aa*^ embryos. *Gdf8*^*Gdf11aa*^ and *Gdf8*^*Gdf11MD*^ embryos all had T13 axial vertebrae and T7 vertebrosternal ribs with 100% penetrance. No additional anomalies outside of these vertebral aberrations were observed. The total number of embryos represents two separate cohorts harvested from multiple F4 mono-allelic *Gdf11*^*Gdf8aa*^, *Gdf8*^*Gdf11aa*^, and *Gdf8*^*Gdf11MD*^ breeding pairs. Percentage of genotypes are shown in parentheses ( ).

We also investigated early-stage craniofacial bone development in *Gdf11* and *Gdf8* mutant mice (Fig 3G and H) because palatal defects have been reported in mice with *Gdf11*^*−/−*^ deletion (McPherron et al, 1999; Cox et al, 2019) and in humans with *Gdf11* loss-of-function alleles (Ravenscroft et al, 2021). In our inspection, the only skeletal differences were detected in the axial vertebral patterning and vertebrosternal rib count of *Gdf11*^*8aa/8aa*^ mutant mice (Fig 3A and C). No defects were noted in the limbs or cranium of *Gdf11*^*Gdf8aa*^, *Gdf8*^*Gdf11aa*^, or *Gdf8*^*Gdf11MD*^ mutants, compared with *Gdf11*^*+/+*^ and *Gdf8*^*+/+*^ mice (Fig 3G and H). We also saw no palatal defects consistent with those previously reported for Gdf11-null mice (McPherron et al, 1999), nor did we observe a hole in the otic capsule of mutant mice, which has been reported in *Gdf11* indel and gene-targeted mice (Goldstein et al, 2019) (Fig 3G and H). These results suggest that although full potency of GDF11 may not be necessary for craniofacial bone development, it is crucial for proper axial skeletal development. Therefore, dampening the potency of mature GDF11 with substitution of GDF8 residues is not compatible with maintaining fully normal developmental function, even with appropriate patterning of expression provided by the endogenous *Gdf11* genomic locus. On the contrary, it appears that increasing the potency of GDF8, even to the maximum level of GDF11, does not elicit malformations detectable in early development. Together, these data underscore the notion that GDF11 and GDF8 are functionally distinct during development.

### *Gdf8*^*Gdf11MD*^ mutants exhibit decreased skeletal muscle mass, whereas the muscles of *Gdf11*^*Gdf8aa*^ and *Gdf8*^*Gdf11aa*^ mutants are not significantly altered

**Figure 4.**
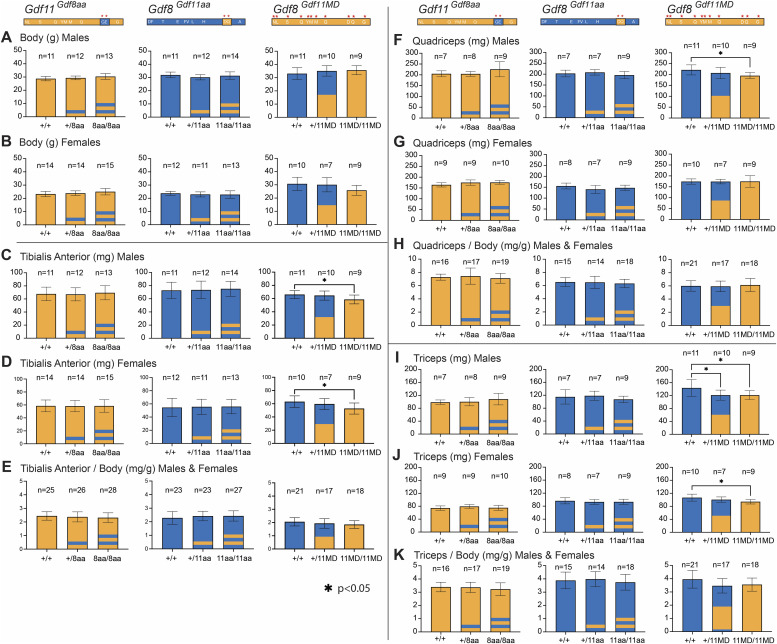
Body weight and muscle mass in *Gdf11*^*Gdf8aa*^, *Gdf8*^*Gdf11aa*^, and *Gdf8*^*Gdf11MD*^ mice. Tibialis anterior, quadriceps, and triceps muscles were collected postmortem from *Gdf11*^*Gdf8aa*^, *Gdf8*^*Gdf11aa*^, and *Gdf8*^*Gdf11MD*^ mice at 10–14 wk of age. **(A, B)** Male and (B) female mice were weighed before dissection. **(C, D, E)** Weights of the tibialis anterior muscle in (C) male and (D) female mice were normalized to overall body weight (E) and showed a statistically significant decrease in both male and female *Gdf8*^*11MD/11MD*^ mutants, compared with *Gdf8*^*+/+*^. **(F, G, H, I, J, K)** Quadriceps weights in (F) male and (G) female mice normalized to body weight (H) showed a significant decrease in male *Gdf8*^*11MD/11MD*^ mice only, compared with *Gdf8*^*+/+*^, whereas the weight of the triceps in (I) male and (J) female mice normalized to body weight (K) showed a significant decrease in both male and female *Gdf8*^*11MD/11MD*^ mice, compared with *Gdf8*^*+/+*^. However, no significant differences were observed for normalized muscle weights in these mutant mice, compared with WT. Statistical analysis was performed by one-way ANOVA with Tukey’s correction for multiple comparisons. Amino acid residues of *Gdf11* are represented in orange, and those of *Gdf8*, in blue. For *Gdf11*^*Gdf8aa*^ and *Gdf8*^*Gdf11aa*^ lines, one stripe and two stripes denote mono-allelic and bi-allelic replacement, respectively. For *Gdf8*^*Gdf11MD*^ mice, half orange denotes mono-allelic replacement, and full orange denotes bi-allelic replacement. Also see Figs S3 and S4.

**Figure S3.**
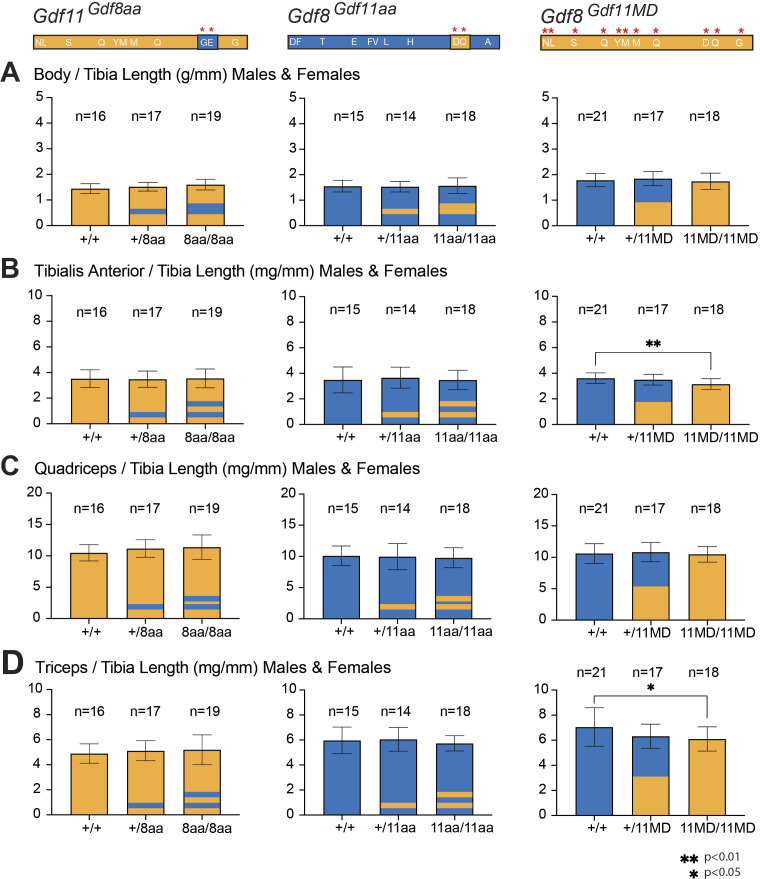
Skeletal muscle measurements normalized to tibia length for *Gdf11*^*Gdf8aa*^, *Gdf8*^*Gdf11aa*^, and *Gdf8*^*Gdf11MD*^ mice. **(A, B, C, D)** Overall body weight (A), tibialis anterior muscle (B), quadriceps muscle (C), and triceps muscle (D) weights of *Gdf11*^*Gdf8aa*^, *Gdf8*^*Gdf11aa*^, and *Gdf8*^*Gdf11MD*^ mice at 10–14 wk of age were normalized to tibia length. **(B, D)** In *Gdf8*^*Gdf11MD*^ mice, there was a statistically significant decrease in the (B) tibialis anterior muscle normalized to tibia length between *Gdf8*^*11MD/11MD*^ (n = 18) versus *Gdf8*^*+/+*^ (n = 21, *P* < 0.01) mice, and in the (D) triceps muscle normalized to tibia length between *Gdf8*^*11MD/11MD*^ (n = 18) versus *Gdf8*^*+/11MD*^ (n = 21, *P* < 0.05) mice. Statistical analysis was performed by one-way ANOVA with Tukey’s correction for multiple comparisons.

**Figure S4.**
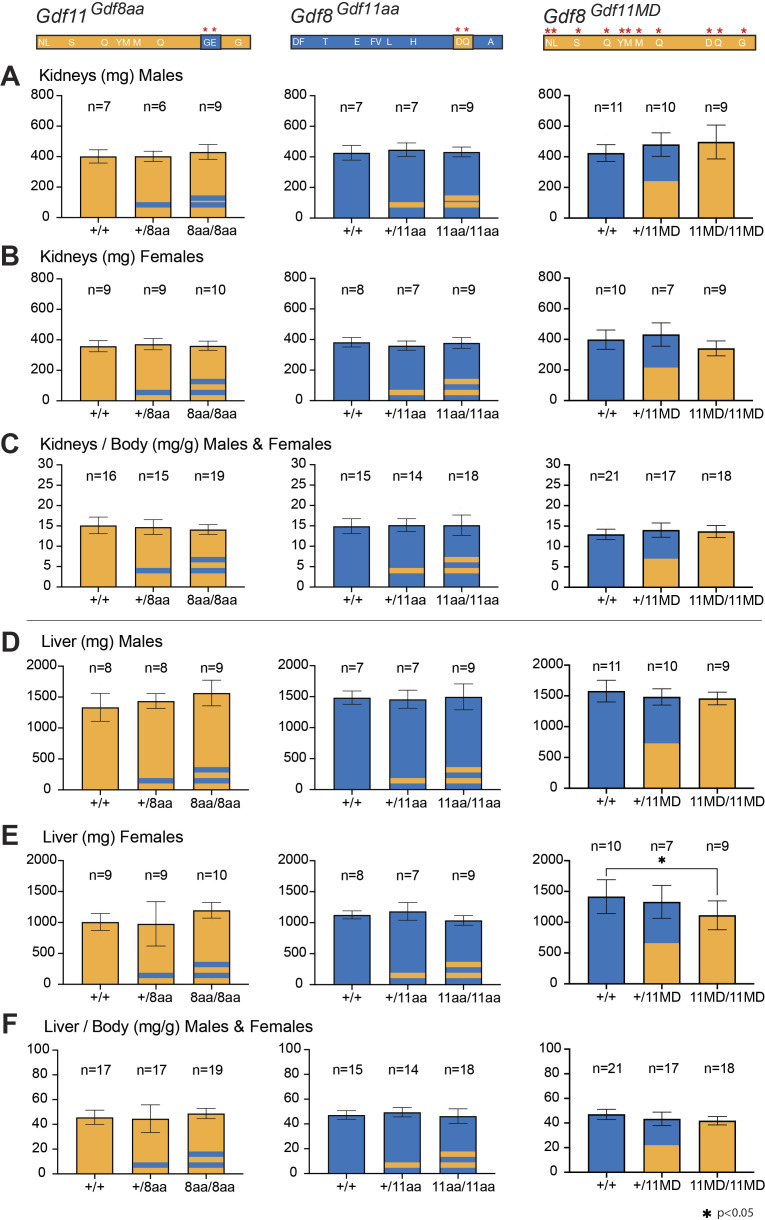
Kidney and liver weights of *Gdf11*^*Gdf8aa*^, *Gdf8*^*Gdf11aa*^, and *Gdf8*^*Gdf11MD*^ mice. **(A, B, C)** Kidney weights (from two kidneys) of *Gdf11*^*Gdf8aa*^, *Gdf8*^*Gdf11aa*^, and *Gdf8*^*Gdf11MD*^ (A) male and (B) female mice were taken at 10–14 wk of age and normalized to overall body weight (C). No mutant mouse of any chimeric line was represented with fewer than two kidneys. In *Gdf11*^*Gdf8aa*^, *Gdf8*^*Gdf11aa*^ mice, no significant differences in kidney weights were found in mutants, compared with WT. **(D, E, F)** Similarly, the liver weight from (D) male and (E) female mice was normalized to body weight (F). **(E)** Significant decrease was found in the liver weight of (E) female *Gdf8*^*11MD/11MD*^ mutants (n = 9), compared with *Gdf8*^*+/+*^ mice (n = 10, *P* < 0.05). **(D, F)** However, the corresponding difference was not found in the liver weight of the males (D) or in the normalized weight of male and female *Gdf8*^*Gdf11MD*^ mice (F). Statistical analysis was performed by one-way ANOVA with Tukey’s correction for multiple comparisons.

Next, we examined early-stage skeletal muscle and cardiac development in the *Gdf11*^*Gdf8aa*^, *Gdf8*^*Gdf11aa*^, and *Gdf8*^*Gdf11MD*^ mouse lines. Prior studies indicate that genetic inactivation of *Gdf8* dramatically increases muscle mass and alters fiber-type distribution across multiple animal species and in a dose-dependent manner (McPherron et al, 1997; McPherron & Lee, 1997), whereas boosting levels of GDF8 protein has been shown to drive muscle wasting (Zimmer et al, 2002; Artaza et al, 2007; Stolz et al, 2008). We therefore sought to determine whether enhancing the potency of mature GDF8 in the *Gdf8*^*Gdf11aa*^ or *Gdf8*^*Gdf11MD*^ mutants might reduce muscle mass compared with *Gdf8*^*+/+*^ mice, harvesting and analyzing the wet weight of the tibialis anterior (TA) (Fig 4C and D), quadriceps (Fig 4F and G), and triceps (Fig 4I and J) muscles postmortem across all three chimeric lines at 10–14 wk of age. Muscle weights were normalized to both total body weight (Fig 4A and B) and tibia bone length (Fig S3) in the *Gdf11*^*Gdf8aa*^, *Gdf8*^*Gdf11aa*^, and *Gdf8*^*Gdf11MD*^ mutants (Fig 4E, H, and K) to assess possible changes in muscle mass. In addition, we harvested and weighed the kidneys from each mouse and normalized them to total body weight (Fig S4).

Across the chimeric amino acid *Gdf8*^*Gdf11aa*^ and *Gdf11*^*Gdf8aa*^ lines, we saw no significant differences in body weight (Fig 4A and B), TA (Fig 4C and D), quadriceps (Fig 4F and G), or triceps weights (Fig 4I and J). Skeletal muscle weights normalized to total body weight (Fig 4E, H, and K) and to tibia length (Fig S3B–D) were indistinguishable. Overall body weight normalized to tibia length was also insignificant in these two lines (Fig S3A). Across the *Gdf8*^*Gdf11MD*^ mutants, however, the weights of these skeletal muscles were significantly decreased (Fig 4C, D, F, I, and J). Notably, TA (Fig 4C), quadriceps (Fig 4F), and triceps (Fig 4I) weights all were reduced to a statistically significant level in bi-allelic *Gdf8*^*11MD/11MD*^ mutant males (n = 9, *P* < 0.05), compared with sex-matched *Gdf8*^*+/+*^ mice (n = 10), with similarly significant decreases recorded in TA (Fig 4D) and triceps (Fig 4J) weights in *Gdf8*^*11MD/11MD*^ mutant females (n = 9, *P* < 0.05) as well. Although all three muscle groups in *Gdf8*^*11MD/11MD*^ mice normalized to overall body weight did not yield statistically significant differences (Fig 4E, H, and K), the raw weights of TA muscle normalized to tibia length (n = 18, *P* < 0.01) (Fig S3B) and triceps muscle normalized to tibia length (n = 18, *P* < 0.05) (Fig S3D) showed statistically significant decreases, compared with *Gdf8*^*+/+*^ mice (n = 21). Overall, we did not observe any distinct defects or malformations in muscle development or patterning at any point during development, and the chimeric mice appeared similar to WT into early adulthood. In all cases, separation by males and females resulted in shifts in the mean muscle mass between the sexes. Analysis of muscle mass in *Mstn*^*Gdf11/Gdf11*^ mice showed similar (∼10%) reductions in muscle mass in male, but not female, mice in which the mature domain of GDF8 was replaced by that of GDF11, with no differences in fiber composition (Lee et al, 2022). However, these studies did not include analysis of the dual amino acid substitutions reported here.

Although GDF11 loss of function frequently leads to kidney agenesis (Esquela & Lee, 2003; McPherron et al, 2009), we did not observe any chimeric mouse lacking a kidney. The combined weight of both kidneys (Fig S4A–C) also did not show any significant change in *Gdf11*^*Gdf8aa*^, *Gdf8*^*Gdf11aa*^, and *Gdf8*^*Gdf11MD*^ mutants, compared with WT mice. Interestingly, we did record a statistically significant decrease in the liver weight of *Gdf8*^*11MD/11MD*^ females (n = 9), compared with *Gdf8*^*+/+*^ females (n = 10, *P* < 0.01) (Fig S4E), but a similarly significant decrease was not found in *Gdf8*^*Gdf11MD*^ mutant males (Fig S4D), despite a modest, yet progressive, weight decline in the liver of *Gdf8*^*+/11MD*^ mutants, followed by *Gdf8*^*11MD/11MD*^ mutants, compared with *Gdf8*^*+/+*^ mice (Fig S4D–F). This trend was not seen in *Gdf8*^*Gdf11aa*^ mice (Fig S4D–F).

Taken together, these data indicate that the increased potency conferred to GDF8 by substitution of the GDF11 mature domain significantly impacted skeletal muscle mass in young *Gdf8*^*Gdf11MD*^ mutant mice, in line with prior reports that endogenous GDF8 negatively regulates muscle development. The observed effects were distinct from those conferred to young *Gdf8*^*Gdf11aa*^ mutant mice by substitution of only the *Gdf11*-like 89/91 residues—which did not yield any significant change in skeletal muscle mass. These data indicate a clear difference in potency between the mature domains of GDF11 and GDF8, and between the full GDF11 mature domain–replaced ligand and the double amino acid–substituted ligand generated here. In addition, though we did not observe signs of kidney agenesis in the *Gdf8*^*Gdf11MD*^ mouse line, we recorded a trending decline in liver weight, most significant in *Gdf8*^*Gdf11MD*^ mutant females. These data suggest a possible systemic effect on the liver—which does not produce GDF8—that could reflect a direct effect on liver hepatocytes or, more likely, be connected to the local GDF8 regulation of skeletal muscle mass. In such a scenario, skeletal muscle may be more sensitive to increased potency of GDF8, and as a result, a change in muscle mass indirectly affects liver development or homeostasis.

### Baseline cardiac physiology and function remain unchanged in *Gdf11*^*Gdf8aa*^, *Gdf8*^*Gdf11aa*^, and *Gdf8*^*Gdf11MD*^ mutants

**Figure 5.**
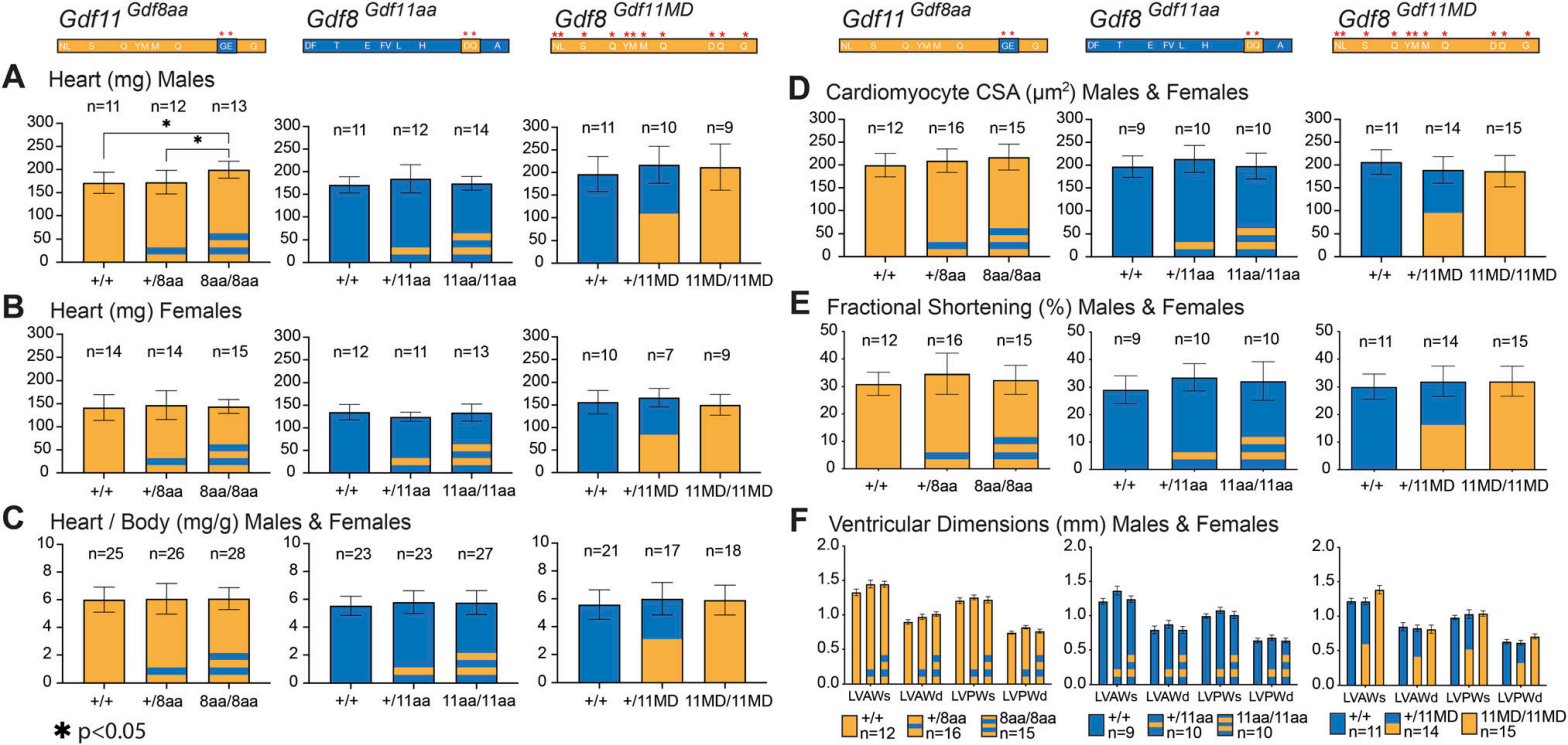
Baseline heart physiological and functional measurements in *Gdf11*^*Gdf8aa*^, *Gdf8*^*Gdf11aa*^, and *Gdf8*^*Gdf11MD*^ mice. **(A, B, C)** Hearts of *Gdf11*^*Gdf8aa*^, *Gdf8*^*Gdf11aa*^, and *Gdf8*^*Gdf11MD*^ (A) male and (B) female mice were harvested and weighed at 10–14 wk of age and normalized to overall body weight (C). **(D, E, F)** Cardiomyocyte cross-sectional area (D), fractional shortening (E), and left ventricular heart dimensions (LVAW; LVPW; LVID) (F) during systole and diastole were measured across all three mouse lines. **(A)** Statistically significant increase in heart weight was found in (A) *Gdf11*^*8aa/8aa*^ males (n = 13), compared with *Gdf11*^*+/+*^ (n = 11, *P* < 0.05) and with *Gdf11*^*+/8aa*^ males (n = 12, *P* < 0.05). **(B, C)** However, no similar difference was observed in (B) *Gdf11*^*Gdf8aa*^ females, and the overall weight of *Gdf11*^*Gdf8aa*^ mutant hearts normalized to body weight (C) did not yield a significant difference either. The cross-sectional area of cardiomyocytes was measured using FIJI software (scale bar = 100 *µ*m). M-mode was used to measure left ventricular interventricular septal wall thickness (IVS/LVAW), left ventricular posterior wall thickness, and left ventricular internal diameter. Statistical analysis was performed by one-way ANOVA with Tukey’s correction for multiple comparisons. Amino acid residues of *Gdf11* are represented in orange, and those of *Gdf8*, in blue. Also see Fig S5.

**Figure S5.**
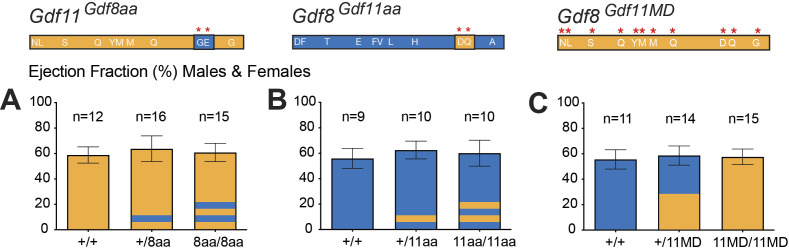
Cardiac ejection fraction readings of *Gdf11*^*Gdf8aa*^, *Gdf8*^*Gdf11aa*^, and *Gdf8*^*Gdf11MD*^ mice. **(A, B, C)** Heart ejection fraction (ES) measurements were taken in (A) *Gdf11*^*Gdf8aa*^, (B) *Gdf8*^*Gdf11aa*^, and (C) *Gdf8*^*Gdf11MD*^ mice and compared across all genotypes of each mouse line. Overall, no significant differences between any genotypes were found. Statistical analysis was performed by one-way ANOVA with Tukey’s correction for multiple comparisons.

Previous studies have shown that administration of exogenous recombinant GDF11 to aged mice reduces cardiac hypertrophy (Loffredo et al, 2013), and fetal cardiac GDF8 has also been implicated in early-stage heart development (Sharma et al, 1999). Since our in vitro experiments showed that GDF11 is a more potent signaling ligand than GDF8, there is potential for the genetically engineered mutants to induce changes in cardiac parameters. Therefore, we harvested and weighed the hearts of all chimeric mice at 10–14 wk of age (Fig 5A and B). In the *Gdf11*^*Gdf8aa*^ line, we found a statistically significant increase in heart weight of *Gdf11*^*8aa/8aa*^ males (n = 13), compared with *Gdf11*^*+/+*^ males (n = 11, *P* < 0.05) and with *Gdf11*^*+/8aa*^ males (n = 12, *P* < 0.05) (Fig 5A). However, this difference was not observed in *Gdf11*^*Gdf8aa*^ female mice (Fig 5B), and the overall weight of *Gdf11*^*Gdf8aa*^ mutant hearts normalized to body weight did not show a significant difference (Fig 5C). Analyses of *Gdf8*^*Gdf11aa*^ and *Gdf8*^*Gdf11MD*^ mouse lines showed no significant difference in the weight of mutant hearts, compared with that of *Gdf8*^*+/+*^ mice (Fig 5A and B). Further histopathological analysis of cardiac tissue also did not reveal significant differences in cardiomyocyte cross-sectional area (CSA) in any mutant lines compared with age- and sex-matched *Gdf11*^*+/+*^ and *Gdf8*^*+/+*^ mice (Fig 5D). We also investigated whether the mutations introduced to native *Gdf11* and *Gdf8* altered baseline cardiac physiology or function by performing blinded echocardiographic studies on all the chimeric mice at 10–14 wk of age (Fig 5E and F). In all three lines, echocardiographic imaging indicated equivalent baseline cardiac function. Fractional shortening (FS) (Fig 5E) and left ventricular heart dimensions (LVAW; LVPW; LVID) during systole and diastole (Fig 5F) were consistent across all genotypes, with no significant differences observed. Cardiac ejection fraction (ES) was also comparable across all three lines (Fig S5A–C).

These results indicate that the *Gdf11*^*Gdf8aa*^, *Gdf8*^*Gdf11aa*^, and *Gdf8*^*Gdf11MD*^ mutant hearts have function and physiology similar to WT mice. Although no heart weight phenotype has been reported for *Gdf11*^*+/−*^ mice, a difference was found in the heart weight of bi-allelic *Gdf11*^*8aa/8aa*^ mutant males (Fig 5A). However, this difference was not present in *Gdf11*^*8aa/8aa*^ female hearts (Fig 5B), and overall, we saw no significant differences across heart physiology and function of *Gdf8*^*Gdf11aa*^ mutants. Furthermore, conferring the full potency of GDF11 to GDF8 did not significantly impact cardiac muscle CSA or ventricular dimensions in young *Gdf8*^*Gdf11MD*^ mutant mice (Figs 5 and S5). If potency changes in either GDF11 or GDF8 indeed regulate cardiac muscle, then they do not appear to do so during development or young adulthood.

### *Gdf11*^*Gdf8aa*^, *Gdf8*^*Gdf11aa*^, and *Gdf8*^*Gdf11MD*^ mice exhibit normal regeneration of damaged muscle after cryoinjury

**Figure 6.**
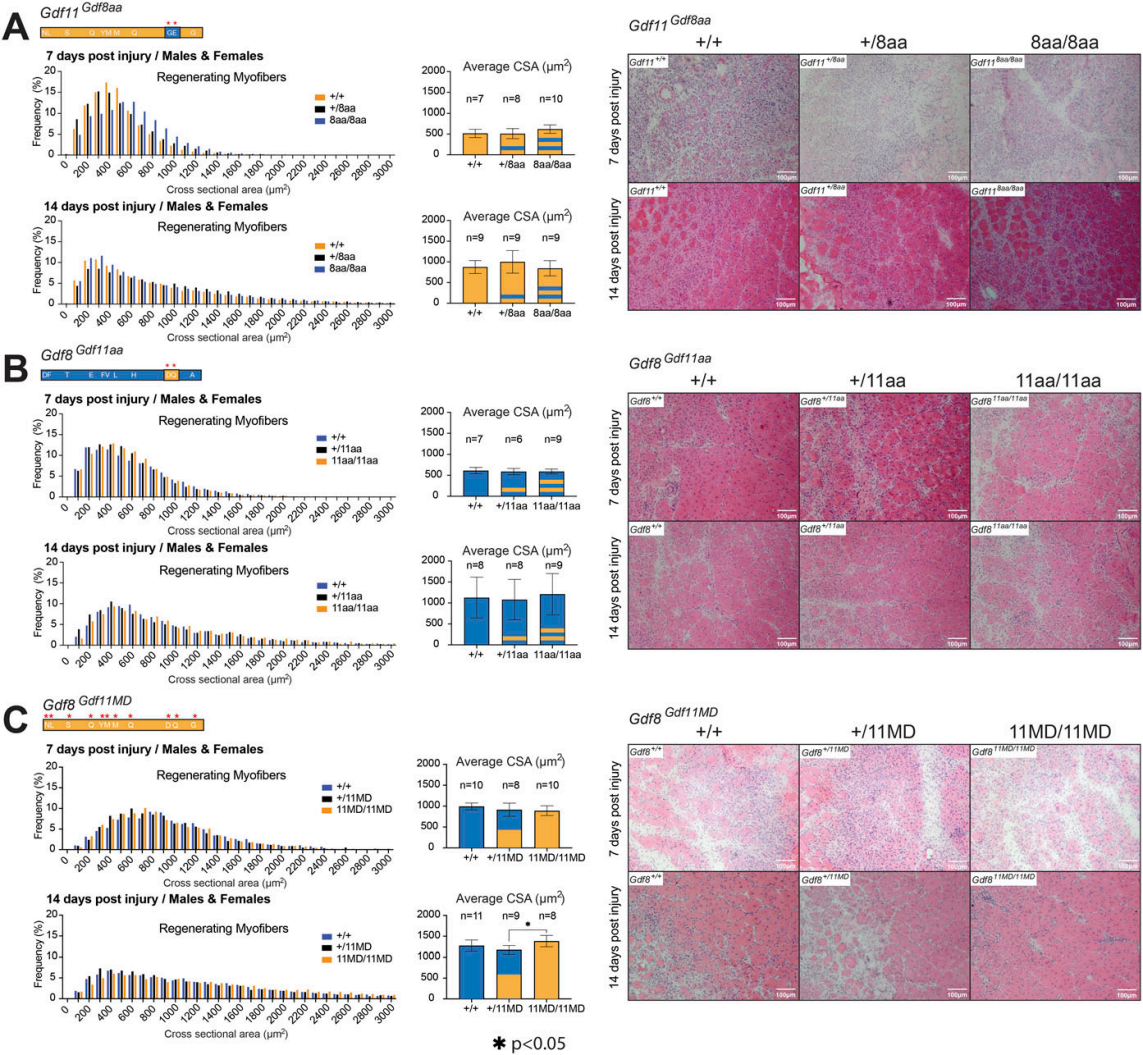
Regeneration in damaged muscle post-cryoinjury in *Gdf11*^*Gdf8aa*^, *Gdf8*^*Gdf11aa*^, and *Gdf8*^*Gdf11MD*^ mice. The tibialis anterior muscles of male and female mice were harvested at 7 and 14 d post-injury and analyzed by H&E staining. **(A, B, C)** Cross-sectional area of the regenerating centrally nucleated fibers within the injured tibialis anterior muscle was measured using FIJI software (scale bar = 100 *µ*m) and compared between (A) *Gdf11*^*Gdf8aa*^, (B) *Gdf8*^*Gdf11aa*^, and (C) *Gdf8*^*Gdf11MD*^ mice at each time point. Overall, no significant differences were found in the frequency or size of regenerating myofibers in any genotype. However, at 14 d post-injury, bi-allelic *Gdf8*^*Gdf11MD/11MD*^ mice exhibited modest increased cross-sectional area compared with *Gdf8*^*+/11MD*^, but not compared with *Gdf8*^*+/+*^ mice. Statistical analysis was performed by one-way ANOVA with Tukey’s correction for multiple comparisons. Also see Fig S6.

**Figure S6.**
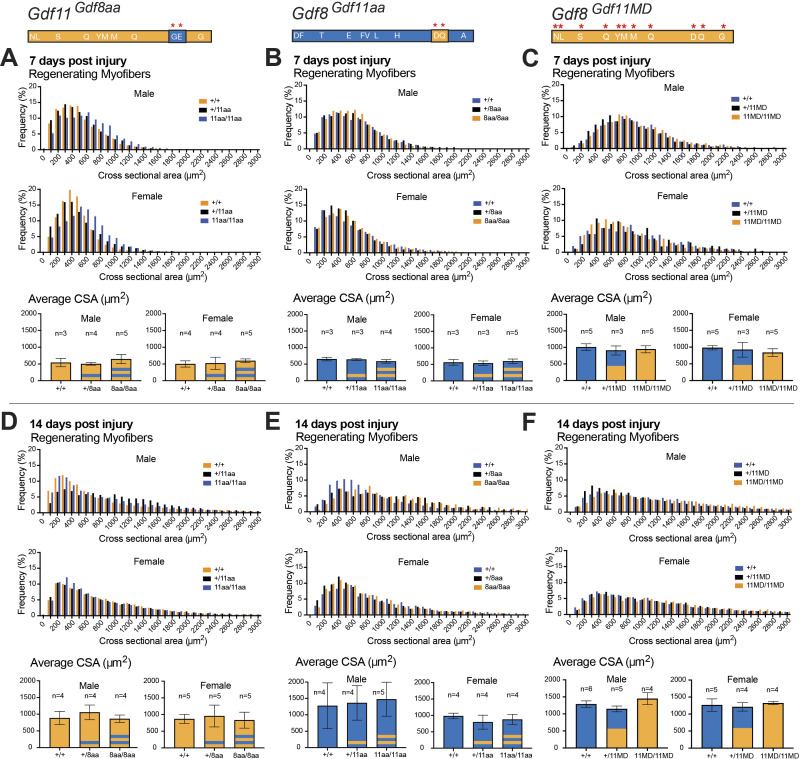
Normal regeneration of damaged muscle in male and female *Gdf11*^*Gdf8aa*^, *Gdf8*^*Gdf11aa*^, and *Gdf8*^*Gdf11MD*^ mice after cryoinjury. **(A, B, C, D, E, F)** Tibialis anterior muscles of male and female *Gdf11*^*Gdf8aa*^, *Gdf8*^*Gdf11aa*^, and *Gdf8*^*Gdf11MD*^ mice were harvested at 7 d (A, B, C) and 14 d (D, E, F) post-injury and analyzed by H&E staining (respectively). The cross-sectional area of regenerating centrally nucleated fibers was measured using FIJI software (scale bar = 100 *µ*m). No statistically significant difference in frequency or size of regenerating myofibers was found between genotypes in either male or female mice of any mutant line. Statistical analysis was performed by one-way ANOVA with Tukey’s correction for multiple comparisons.

It has been reported that muscle repair after toxin-induced injury to skeletal myofibers is significantly enhanced in *Mstn*-null mice (McCroskery et al, 2005; Wagner et al, 2005), suggesting that endogenous GDF8 suppresses satellite cell proliferation. Debate continues as to whether GDF8 acts directly on muscle satellite cells and whether such action may account, at least in part, for the muscle hyperplasia or hypertrophy observed in *Mstn*-null mice (Thomas et al, 2000; Taylor et al, 2001; Amthor et al, 2009; Garikipati & Rodgers, 2012; Lee et al, 2012; George et al, 2013; Walker et al, 2016). To test what impact enhancing GDF8 potency has on muscle regenerative capacity, we subjected *Gdf8*^*Gdf11aa*^ and *Gdf8*^*Gdf11MD*^ mutants to cryoinjury (Fig 6). TA muscles of chimeric mice at 10–14 wk of age were cryoinjured on day 0, harvested at 7 and 14 d post-injury, and analyzed via H&E staining (Figs 6 and S6). Following cryoinjury, previously quiescent satellite cells in the basal lamina of myofibers are activated, giving rise to proliferating myoblasts (Dumont et al, 2015), which further differentiate and fuse together to form myotubes. Newly regenerated fibers can be distinguished by their central nuclei, with larger sized fibers at early time points in the repair process corresponding to more advanced muscle fiber regeneration (Mauro, 1961; Cosgrove et al, 2014; Sinha et al, 2014). We similarly challenged *Gdf11*^*Gdf8aa*^ mutants in muscle regeneration assays, as conflicting reports have been published regarding the impact of changing levels of GDF11 on muscle repair (Sinha et al, 2014; Egerman et al, 2015).

Analysis of regenerating myofibers in cryodamaged muscles (7 or 14 d post-injury) showed no significant differences in average CSA or fiber size distribution in either *Gdf8*^*Gdf11aa*^ or *Gdf11*^*Gdf8aa*^ mice (Figs 6A–D and S6). These data indicate that neither increasing the potency of GDF8 nor dampening that of GDF11 is sufficient to alter the time course or outcome of muscle fiber regeneration in young adult mice. Taken together with the lack of muscle or body weight change in *Gdf8*^*Gdf11aa*^ or *Gdf11*^*Gdf8aa*^ mice, it appears that postnatal skeletal muscle is more tolerant of changes in GDF11 signaling potency than embryonic bone, which exhibited skeletal transformations when GDF11 potency was reduced. In the *Gdf8*^*Gdf11MD*^ mutants, regenerating fiber size in *Gdf8*^*+/11MD*^ and *Gdf8*^*11MD/11MD*^ mutants was indistinguishable from WT at both time points (Fig 6E and F). However, fiber size appeared slightly increased at 14 d post-injury in bi-allelic *Gdf8*^*11MD/11MD*^ mutants compared with mono-allelic *Gdf8*^*+/11MD*^ mutants (Fig 6E). These data raise the possibility that the lack of mature GDF8, or the increased abundance of mature GDF11 protein, or the combination of these events might have a positive impact on the rate of muscle repair, consistent with prior reports of enhanced muscle regeneration after the loss of GDF8 (McCroskery et al, 2005; Wagner et al, 2005) or supplementation of GDF11 in vivo (Sinha et al, 2014).

Altogether, these data support the notion that the modest changes in ligand potency achieved through dual amino acid mutations confer a genetically linked phenotype in early-stage skeletal development within only *Gdf11*^*Gdf8aa*^ mutant mice. In addition, the heightened ligand potency achieved through modifying the full mature domain of GDF8 in *Gdf8*^*Gdf11MD*^ mutants confers a genetically linked phenotype impacting skeletal muscle mass in adult mice. Incredibly, in bi-allelic *Gdf8*^*11MD/11MD*^ mutants, circulating GDF11 concentration increased ∼50-fold more than normal, resulting in unexpectedly high levels of circulating GDF11 in vivo. Yet these chimeric mice were viable and survived normally into adulthood. The results indicate that gene regulation differences or differences in the prodomain regions may influence changes in ligand concentrations in vivo, and that ligand activity may not reflect alterations to ligand potency alone.

## Discussion

### Maintenance of GDF11 ligand potency and function is required for normal skeletal development

In this study, we investigated key phenotypes resulting from changes in the amino acid residues of GDF11 or GDF8, brought on by genetic modifications made to the *Gdf11* or *Gdf8* mature signaling domains in vivo. Results presented here indicate that changes in GDF11 and GDF8 potency elicit differential phenotypes (Table 2). Initial screening in early development revealed abnormal embryonic skeletal and vertebral transformations in the *Gdf11*^*8aa/8aa*^ mutant mice, similar to those of mice with heterozygous (*Gdf11*^*+/−*^) deletion of *Gdf11* (McPherron et al, 1999). We followed these bi-allelic mutants of the *Gdf11*^*Gdf8aa*^ line into adulthood to determine whether dampened GDF11 function in *Gdf11*^*Gdf8aa*^ mutants results in additional phenotypic differences in organ growth and function postnatally. Assessments of overall body condition, skeletal and cardiac muscle mass, serum protein concentration, skeletal muscle repair, and baseline heart physiology and function were performed. Similar studies of the *Gdf8*^*Gdf11aa*^ mutants were done in parallel to determine whether increased GDF8 potency in these mice resulted in differential phenotypes in adulthood. These efforts focused particularly on muscle size and function, given GDF8’s well-established negative regulation of muscle growth (McPherron et al, 1997; McPherron & Lee, 1997; Walker et al, 2016).

**Table 2.** Developmental patterns and phenotypes of *Gdf11*^*Gdf8aa*^, *Gdf8*^*Gdf11aa*^, and *Gdf8*^*Gdf11MD*^ mice.

Tissue/Phenotype	GDF11	GDF8	
Predominant expression pattern	Developing limb buds; primitive streak and tail bud	Development and adult skeletal muscle maintenance	
Chimeric lines	*Gdf11* ^*Gdf8aa*^	*Gdf8* ^*Gdf11aa*^	*Gdf8* ^*Gdf11MD*^
Premature lethality	No	No	No
Bone (E18.5)	Transformation of the axial skeleton (T14 thoracic vertebrae, T8 vertebrosternal ribs)	No difference compared with WT	No difference compared with WT
Circulating ligand concentration (10–14 wk)	No difference compared with WT	No difference compared with WT	∼50-fold increase in GDF11 in bi-allelic mutants; GDF8 levels at or below the level of detection
Adult skeletal muscle (10–14 wk)	No difference compared with WT	No difference compared with WT	Statistically significant decrease in mutant tibialis anterior, quadriceps, and triceps weights
Heart (10–14 wk)	Statistically significant increase in mutant male heart weights; not observed in mutant females	No difference in function and physiology compared with WT	No difference in function and physiology compared with WT
Cardiac myocytes (10–14 wk)	No difference in cross-sectional area compared with WT	No difference in cross-sectional area compared with WT	No difference in cross-sectional area compared with WT
Kidney (10–14 wk)	Normal compared with WT; no observed renal agenesis	Normal compared with WT; no observed renal agenesis	Normal compared with WT; no observed renal agenesis
Liver (10–14 wk)	Observed slight increase in bi-allelic mutant liver weight, however not statistically significant	No difference compared with WT	Statistically significant decrease in bi-allelic mutant female liver weight; not observed in mutant males
Injured muscle regeneration (10–14 wk, harvest at 7 and 14 d post-injury)	No difference compared with WT	No difference compared with WT	No difference compared with WT

Summary of phenotypic outcomes observed in *Gdf11*^*Gdf8aa*^, *Gdf8*^*Gdf11aa*^, and *Gdf8*^*Gdf11MD*^ mice. Skeletal transformations were observed only in *Gdf11*^*Gdf8aa*^ mice, resulting in the addition of one thoracic vertebra (T14 total) and vertebrosternal rib (T8 total) in bi-allelic *Gdf11*^*8aa/8aa*^ mutants. In *Gdf8*^*Gdf11MD*^ mice, bi-allelic *Gdf8*^*11MD/11MD*^ mutants had increased levels of circulating GDF11 ∼50-fold higher than WT mice. *Gdf8*^*Gdf11MD*^ mutants also presented with a statistically significant decrease in skeletal muscle weights at 10–14 wk of age. No mouse lines exhibited premature lethality or showed differences in overall heart physiology and function, kidney agenesis, liver weight, or muscle regeneration 7 and 14 d post-injury.

Our findings show that reducing the potency of mature GDF11 toward that of mature GDF8 is insufficient to sustain normal developmental function. Specifically, exchanging two amino acid residues from the *Gdf8* mature domain (G89 and E91) into the analogous location in the *Gdf11* mature domain (D89 and Q91) decreased the potency of mature GDF11 to that of GDF8 (Walker et al, 2017) and resulted in skeletal transformations detectable during early development in *Gdf11*^*Gdf8aa*^ mice. However, exchanging the same amino acids from the *Gdf11* locus into the corresponding location in *Gdf8* did not produce similar physiological defects, and *Gdf8*^*Gdf11aa*^ mice carrying one or both chimeric alleles were indistinguishable from WT mice in all of the assays we performed.

Because both GDF8 and GDF11 have been shown to play critical roles in skeletal and cardiac muscle development and function, and to regulate other organ systems (McPherron et al, 1997; Lee & Lee, 2013; Walker et al, 2016), further experiments were conducted to investigate the phenotypes of *Gdf11*^*Gdf8aa*^ and *Gdf8*^*Gdf11aa*^ mutant mice in early adulthood. Despite the axial skeletal defects found in the *Gdf11*^*Gdf8aa*^ line, reduction in GDF11 potency in these mutants did not impact postnatal skeletal muscle growth or regenerative activity, nor did it alter baseline cardiac physiology or function into adulthood. We observed no differences in body weight or skeletal muscle weight (TA, quadriceps, and triceps muscle; normalized to body weight and tibia length) postnatally in male or female mutants at 10–14 wk of age. Likewise, *Gdf8*^*Gdf11aa*^ mice did not show significant differences in either sex at 10–14 wk of age. For both chimeric lines, we saw no differences in heart or kidney weight in either the *Gdf11*^*Gdf8aa*^ or *Gdf8*^*Gdf11aa*^ mutants. The only statistically significant finding occurred in the heart weight of *Gdf11*^*8aa/8aa*^ mutant males, compared with *Gdf11*^*+/+*^ and *Gdf11*^*+/8aa*^ mice; however, this result was not observed in mutant females of the same line. The differences in the heart weight normalized to body weight also proved insignificant in the *Gdf11*^*8aa/8aa*^ mutants. Interestingly, *Gdf8*^*Gdf11aa*^ mutants did not exhibit physiological changes to those found in *Gdf11*^*Gdf8aa*^ mutants during embryonic development, nor did they produce measurable anatomic differences into adulthood.

### Replacement of GDF8 mature domain with GDF11 decreases skeletal muscle mass and produces a 50-fold increase of circulating GDF11 levels in young adult mutants

Similar to *Gdf8*^*Gdf11aa*^ mutants, chimeric *Gdf8*^*Gdf11MD*^ mice also did not exhibit detectable transformations during embryonic skeletal development, either in mono-allelic *Gdf8*^*+/11MD*^ or in bi-allelic *Gdf8*^*11MD/11MD*^ mutants. Furthermore, we detected neither cranial bone malformation nor cleft palates in the mutants. Therefore, raising the activity of GDF8 to the full potency of GDF11 did not produce measurable changes in osteogenesis. This result may reflect the different tissue-specific expression patterns of the *Gdf11* and *Gdf8* loci. We performed additional experiments to determine whether increased GDF8 function in *Gdf8*^*Gdf11MD*^ mutants might result in phenotypic differences in muscle growth and function postnatally and into adulthood. In *Gdf8*^*Gdf11MD*^ mice, we confirmed that the increase in the GDF8 potency produced by replacing the mature domain of *Gdf8* with that of *Gdf11* decreased skeletal muscle size/mass in several limb muscles, consistent with results from GDF8 supplementation studies (Zimmers et al, 2002; Stolz et al, 2008) and with the recently reported *Mstn*^*Gdf11/Gdf11*^ mice (Lee et al, 2022). Specifically, *Gdf8*^*Gdf11MD*^ mutants exhibited decreased weight of the TA and triceps muscles in early postnatal life in both male and female mice. A significant decrease in quadriceps muscle mass was also noted in mutant males, with bi-allelic mutants exhibiting the greatest change.

Our *Gdf8*^*Gdf11MD*^ mutants also presented a striking outcome in terms of circulating levels of GDF11, which were increased to ∼50-fold more than normal, similar to the 30–40-fold increase in circulating GDF11 protein reported for *Mstn*^*Gdf11/Gdf11*^ mice (Lee et al, 2022). Although some prior studies suggested that elevation of GDF11—even at moderate levels—could result in detrimental consequences in mice, including severe cachexia and premature death (Egerman et al, 2015; Glass, 2016; Schafer et al, 2016; Harper et al, 2018), these results demonstrate that substantial elevation of GDF11 is well tolerated, with only minor effects on skeletal muscle mass. *Gdf8*^*Gdf11MD*^ chimeras were viable into adulthood and showed no signs of premature aging or other negative impacts on health or survival. The profound differences in circulating ligand levels in these gene-modified mice also suggest that gene regulatory differences encoded within the *Gdf8* and *Gdf11* genomic loci play an important role in determining systemic ligand abundance. Whether the substantial increase in GDF11 levels seen in *Gdf8*^*Gdf11MD*^ mutants shifts the homeostasis of known antagonists or alters interactions with their respective N-terminal prodomains remains to be investigated. Finally, this study provides in vivo evidence supporting the molecular explanation for potency differences between GDF11 and GDF8 derived previously from structural and biochemical studies (Walker et al, 2017). In particular, while amino acid differences at residues 89 and 91 are responsible for much of the difference in potency observed between GDF11 and GDF8 (Walker et al, 2017), the other nine amino acids that distinguish the GDF11 and GDF8 mature domains clearly contribute as well.

We found no significant differences in heart weight or baseline cardiac physiology and function in *Gdf8*^*Gdf11MD*^ mutants. However, whether similar results would be obtained in aged mutants or under conditions of transverse aortic constriction remains a question for future studies. Although supraphysiological elevation of GDF11 has been reported in several studies to impede recovery from muscle injury in young mice (Egerman et al, 2015; Hammers et al, 2017; Jones et al, 2018), and loss of GDF11 signaling in older animals has been suggested to underlie poorer regenerative outcomes with aging (Sinha et al, 2014), we also saw no difference in either the kinetics or ultimate outcome of muscle repair after injury in *Gdf8*^*Gdf11aa*^ or *Gdf11*^*Gdf8aa*^ mutants. With these data, we show that robust muscle repair activity is preserved in young mice both when GDF11 signaling is dampened and when GDF11 levels are raised, suggesting that muscle satellite cells and myofibers may be buffered to some extent against changes in GDF11 activity in young adulthood. The only other statistically significant finding occurred in the liver weight of *Gdf8*^*11MD/11MD*^ mutant females, compared with *Gdf8*^*+/+*^ mice and *Gdf8*^*+/11MD*^ mutants, but not in that of *Gdf8*^*11MD/11MD*^ mutant males. At this time, we cannot differentiate whether increased GDF8 potency directly affected liver hepatocytes, leading to the observed change in organ size, or whether indirect effects, potentially linked to changes in skeletal muscle or other tissues, underlie this result. Gene expression analysis in the liver to examine whether regulation of metabolic genes is altered will be useful to verify the possible impact of the change in ligand potency.

Our chimeric mice, genetically modified to change unique amino acid residues between GDF11 and GDF8, demonstrate that sequence-determined structural differences in these ligands are critically important, and not simply accounted for by gene regulation differences alone. We have discovered that two specific amino acids in the fingertip region of the GDF11 mature domain are required for proper axial skeletal patterning during early-stage development, but do not appear to be crucial for regulating heart or skeletal muscle. In addition, substituting the full mature domain sequence of GDF11 into the *Gdf8* locus, in place of mature GDF8, created a GDF8-null mouse with decreased skeletal muscle mass. Circulating GDF11 concentrations in this mutant were significantly higher than WT, showing that very high GDF11 blood levels can be tolerated and can overcome phenotypes typically associated with loss of GDF8 function. Our findings show that changing the ligand potency of GDF11 and GDF8 or altering their bioavailability within the mammalian system causes distinct measurable physiological effects, and that maintenance of functional GDF11 and GDF8 is necessary for proper development and adult tissue maintenance.

## Materials and Methods

### Mouse caretaking

Mouse handling and experimentation followed guidelines set forth by the Institutional Animal Care and Use Committee at Harvard University/Faculty of Arts and Sciences. Regular animal housing and care were carried out by the staff in Biological Laboratories in accordance with relevant Institutional Animal Care and Use Committee regulations and guidelines. Housing included density of two to four mice per cage, along with Enviro-Dri bedding, cotton nestlet, one red hut, automatic waterspout dispensing reverse osmosis/deionized water, and regular chow diet (Prolab IsoPro RMG3000 5P75/76).

### Generation of mutant mouse lines

The CRISPR/Cas9 system was used to generate chimeric mice carrying amino acid mutations from the GDF8 and GDF11 interchanged in the mature domain. Two rounds of microinjections into C57BL/6J zygotes were performed for each line, delivering (1) purified *S. pyogenes* Cas9 mRNA, (2) in vitro–transcribed synthetic guide RNAs (sgRNAs), which targeted the native loci near the desired integration sites, and (3) the generated *Gdf11* and *Gdf8* ssDNA or *Gdf11* dsDNA donor template. Zygote injections were performed by the Genome Modification Facility at Harvard University. Super-ovulated C57BL/6J female mice were mated to C57BL/6J males, and the fertilized zygotes were subsequently harvested from the oviducts. Zygotic pronuclei were microinjected with (1) purified *Streptococcus pyogenes* Cas9 mRNA (100 ng/*µ*l; System Bioscience), (2) in vitro–transcribed synthetic guide RNAs, which targeted near the integration sites of the native loci, and (3) the generated *Gdf11* and *Gdf8* ssDNA donor construct. Microinjected zygotes were implanted into the oviducts of C57BL/6J surrogate females at 12 h post-coitum. To identify mosaic offspring that contained the desired chimeric *Gdf11* and *Gdf8* double amino acid–substituted sequences, progeny from the microinjected surrogate females were genotyped by Sanger sequencing and by PCR validation and subcloning. In the *Gdf11* amino acid chimera, we produced 47 total pups, with 14 live pups and 33 dead pups. Of the 14 live pups, 4 exhibited a mosaic genotype, and 3 were ultimately selected as germline founders. In the *Gdf8* amino acid chimera, we obtained 92 viable pups, with 0 dead pups. Of the 92 live pups, 49 showed a mosaic genotype upon screening for the targeted allele. Ultimately, four confirmed positive founders were used as breeders to establish the colony. In the *Gdf8* full mature domain chimera, we obtained 80 viable pups, with two dead pups. Of the 80 live pups, 35 showed a mosaic genotype upon screening for the targeted allele. Ultimately, five confirmed positive founders were used as breeders to establish the colony. We bred these chosen mice with WT C57BL/6J mice and genotyped the resultant pups to confirm that our desired mutation was incorporated in the germline. We backcrossed the five-generation knock-in alleles in a C57BL/6J genetic background before characterization experiments. Male and female mice were selected based on gender and randomized before treatment for all proposed animal studies.

### Genotyping

Initial mouse colony breeder genotypes were verified by sequencing. Subsequent progeny tissues were collected from the tail or the ear, and mixed in 1.5-ml Eppendorf tubes containing 300 *µ*l of lysis solution (50 mM KCl, 10 mM Tris–HCl, pH 8.3, 2.5 mM MgCl_2_, 0.1 mg/ml gelatin, 0.45% NP-40, and 0.45% Tween-20 in ddH_2_O) with 10 *µ*g/ml Proteinase K and incubated at 50°C for 10–12 h. Next, samples were placed in heating blocks at 98°C for 5 min to inactivate the Proteinase K. DNA was extracted using a standard phenol:chloroform/ethanol precipitation protocol, and genotyping was performed by PCR using Phusion High-Fidelity PCR Master Mix with HF Buffer. Primers used are as follows: for *Gdf11*^*Gdf8aa*^, FW: CCTGACCCTCAGCATCCTTTCA, RV: GGTCCTTACTTTGCCCCATCCT; and for *Gdf8*^*Gdf11aa*^ and *Gdf8*^*Gdf11MD*^, FW: TGTGGTTGGTTTGTTTGTTTGT, RV: GCCTGTGGTGCTTGAATTCA. PCR products were digested with restriction enzyme *AseI* (R0526; New England Biolabs), with NEBuffer 3.1, at 37°C for 20–30 min, and analyzed on 1% agarose gel. Further genotyping was performed via Sanger sequencing to verify the nucleotide changes and the presence of the *AseI* site.

### TLA sequencing

Bone marrow from *Gdf11*^*Gdf8aa*^, *Gdf8*^*Gdf11aa*^, and *Gdf8*^*Gdf11MD*^ F5 mutant mice was harvested, homogenized, and subjected to ACK lysis. Harvested bone marrow (five vials, each containing 1 × 10^7^ cells) was frozen and delivered to Cergentis B.V. for TLA sequencing analysis (de Vree et al, 2014). TLA sequencing used a locus-specific sequence for the targeted amplification and complete sequencing of *Gdf11* and *Gdf8* loci. The genomic DNA was cross-linked, digested, and re-ligated, before it was purified, and circular TLA fragments were then amplified with two independent sets of inverse primers, corresponding to the *Gdf11* or *Gdf8* locus-specific transgene, to identify the location of each targeting event across the whole genome. Primer sets used are as follows:

*Gdf11*^*Gdf8aa*^: Upstream, Fw: ACATTTGCTCCCATTACTGT, Rv: AGCAATAAGAACAAGGGAGC, Downstream, Fw: CAAGAGTCTTAAGAGGATGGG, Rv: GGGTAGTTTAGTAGCTCTCATAG.

*Gdf8*^*Gdf11aa*^: Upstream, Fw: GAATAGATGCAATGGTTGGC, Rv: AGAGTGTAGTGTTTAAGTAGCA, Downstream, Fw: CACAATTTGTTTATGCGGTTT, Rv: TCTCACTTCCTTGCCTAGAT.

*Gdf8*^*Gdf11MD*^: chr10 detection, Fw: CAAGTGGGTGTGTGGATAC, Rv: CTACCAAGATGTCCCCAATC, chr1 detection, Fw: GTAACTGCTCAGATTCCCAA, Rv: AGCTATTCCAAGGAACAACA. 5′ integration site: chr1:53,066,297 (tail) fused to Insert: 1, head (the same as chr10:128,885,435, tail) with four inserted bases ATCCCTTTTTAGAAGTCAAGGTGACAGACACACCCAAGAGGTCCCGAAGAAACCTAGGCCTGGACTGGATGAACACTCGAGTGAGTCCCGCTGCTGCCGATATCCTCTCACAGTGGACTTTGAGGCTTTTGGCTGGACTGGATCATC; 3′ integration site: Insert: 327, tail (the same as chr10:128,885,108, head) fused to chr1:53,066,629 (head) AACATGCTCTACTTCAATGACAAGCAGCAGATTATCTACGGCAAGATCCCTGGCATGGTGGTGGATCGATGTGGCTGCTCCTGAGCTTTGCATTAGGTTAGAAATTTTCCAAGTCATGGAAGGTCTTC. After amplification of the targeted locus, PCR amplicons of the complete *Gdf11* and *Gdf8* region of interest were purified and prepped for Illumina sequencing. Analysis was performed by aligning mutant sequences to the mouse mm10 reference genome sequence. In *Gdf11*^*Gdf8aa*^ mice, two mutated nucleotides (G→C and T→C) were confirmed on chr10 in the mature domain of *Gdf11*, resulting in alteration of only the two targeted amino acids: *Gdf11* D89 to G89 (Asp→Gly) and Q91 to E91 (Gln→Glu). In *Gdf8*^*Gdf11aa*^ mice, two mutated nucleotides (G→A and G→C) were confirmed on chr1 in the mature domain of *Gdf8*, resulting in amino acid changes in *Gdf8* G89 to D89 (Gly→Asp) and E91 to Q91 (Glu→Gln). In bi-allelic *Gdf11*^*8aa/8aa*^ and *Gdf8*^*11aa/11aa*^ samples, the mutated nucleotides were confirmed at 100% frequency, indicating that the mutations occurred on both alleles, whereas in mono-allelic *Gdf11*^*+/8aa*^ and *Gdf8*^*+/11aa*^ samples, the mutations were confirmed at ∼50% frequency, indicating occurrence of the desired mutations on only one allele. In the *Gdf8*^*Gdf11MD*^ mice, correct integration of exon 3 of *Gdf11* was confirmed on chr1 in place of the native exon 3 of *Gdf8*, indicating successful replacement of the GDF8 mature domain with that of GDF11. In bi-allelic *Gdf8*^*11MD/11MD*^ samples, no WT reads were present at the integration site and in the deleted region, confirming bi-allelic replacement of native GDF8, whereas in mono-allelic *Gdf8*^*+/11MD*^ samples, WT reads were detected at the integration site and in the deleted region on one allele, confirming mono-allelic replacement of native GDF8.

### Serum mass spectrometry of GDF11 and GDF8

For serum collection, after euthanasia, blood was collected via orbital bleeding into Microtainer tubes with a serum separator (BD) and incubated for 30 min at room temperature before centrifugation at 2,000*g* for 10 min at room temperature. The upper layer of serum was then transferred to a new tube and stored at −80°C before mass spectrometry analysis. Serum from chimeric mice (minimum 100 *µ*l) was submitted to the Brigham and Women’s Hospital Brigham Research Assay Core (BRAC) for quantitative liquid chromatography–tandem mass spectrometry detection of GDF11 and GDF8 protein concentrations. The mouse serum was denatured and alkylated, followed by pH-based fractionation, using cation ion exchange SPE. After desalting and concentrating, the peptide mix was separated via liquid chromatography, followed by mass spectrometry analysis in a positive electrospray ionization mode. GDF11 and GDF8 concentrations were determined using unique proteotypic peptides from GDF11 and GDF8 as surrogate peptides, coupled with heavy-labeled unique peptides as internal standards. Included in the analysis were GDF8 concentrations and the mean GDF11 concentrations.

### Skeletal preparation

Embryonic day 18.5 (E18.5) harvests (Lewandowski et al, 2019) were performed for skeletal and vertebral analyses of *Gdf11*^*Gdf8aa*^ and *Gdf8*^*Gdf11aa*^ mutants and compared with WT mice. For timed breeding, a vaginal plug observed in the female indicated embryonic day 0.5 (E0.5). Mouse embryos were harvested at E18.5, skinned, and eviscerated, and underwent washes of 100% ethanol and 100% acetone for 24 h at room temperature. The skeletons were then stained using a 0.3% Alcian blue (dyes bone) and 0.1% Alizarin red (dyes cartilage) solution at 37°C while oscillating for 72 h. Next, they were transferred to 1% KOH for 24 h on a rocker at room temperature, followed by a series of glycerol/KOH washes at (1) 20% glycerol/1% KOH, (2) 50% glycerol/1% KOH, and (3) 80% glycerol/1% KOH for 24 h at room temperature. The stained preparations were placed in 80% glycerol/1× PBS and imaged using a Nikon D750 camera attached to a Nikon SMZ1500 stereo microscope with an HR Plan APO 1× objective lens. Images were captured in NEF (Nikon Electronic Format) and processed and adjusted in the Adobe Camera Raw platform.

### Tissue collection and analysis

Mice were ear-tagged and their genotype blinded at 10–14 wk of age. Adult mice were euthanized with CO_2_, and overall body weights were taken postmortem. For tissue collection, the heart was dissected, washed in PBS, and dried on paper towels before weighing. The TA muscle, quadriceps muscle, and triceps muscle were harvested and weighed. The tibia bone was excised, cleaned, and measured using electronic calipers. Statistical analysis was performed using Prism 8.4.2 for macOS. The muscle weights were divided by sex and genotype within each mouse line, and the results were represented as the mean within each group by genotype and sex. The combined muscle weights were normalized to body weight and tibia length. Groups more than two were compared by one-way ANOVA with Tukey’s correction for multiple comparisons.

### Echocardiographic studies

*Gdf11*^*Gdf8aa*^ mice (*Gdf11*^*+/+*^, n = 12; *Gdf11*^*+/8aa*^, n = 16; and *Gdf11*^*8aa/8aa*^, n = 15) and *Gdf8*^*Gdf11aa*^ mice (*Gdf8*^*+/+*^, n = 9; *Gdf8*^*+/11aa*^, n = 10; and *Gdf8*^*11aa/11aa*^, n = 10), age 10–14 wk, were sedated with 0.1–0.5% inhaled isoflurane for echocardiography (Loffredo et al, 2013). Mice were placed on a heating pad, and echocardiograms were obtained at mid-papillary level with the Vevo 3100 (VisualSonics). The heart rate of every mouse studied was monitored and maintained at >400 bpm during echocardiographic procedure, so as to (1) avoid artificial myocardial depression brought on by exposure to isoflurane during imaging and (2) ensure consistent measurements across all study groups. Parasternal long-axis views, short-axis views, and two-dimensional M-mode were used to measure left ventricular interventricular septal wall thickness (IVS/LVAW), left ventricular posterior wall thickness (LVPW), and left ventricular internal diameter (LVID) during both systole and diastole in both mouse lines. Fractional shortening (FS%) was calculated with the VisualSonics software package. Statistical analysis was performed using Prism 8.4.2 for macOS. For echocardiographic and morphometric analyses, CSA, capillary density, and volume results were presented as the mean for each group by genotype and sex. At least three measurements were averaged and used for every data point from each mouse. Groups more than two were compared by one-way ANOVA with Tukey’s correction for multiple comparisons. All analyses were performed under blinded conditions.

### Muscle cryoinjury

The muscle cryoinjury procedure (Oh et al, 2016) was chosen because of its ability to generate a reproducible injury area with a discrete border between injured and uninjured muscles. This border remains distinct during regeneration of injured muscle. Mice were anesthetized using isoflurane, and the skin over the left TA muscle was shaved and disinfected using Betadine, followed by wiping with 70% ethanol. The TA was then exposed by a small 3-mm incision. A metal probe with flat round bottom cooled down in dry ice was applied directly to exposed TA muscle for 5 s. The skin incision was then closed with synthetic absorbable suture (5-0 coated Vicryl) immediately after the injury. Buprenorphine (0.05–0.1 mg/kg, s.c.) was administered immediately after recovery from surgery, and subsequently every 8–12 h, for at least 48 h after surgery. Injured muscles were recovered for 7 or 14 d post-injury. The cryoinjury model employed has been widely used to assess muscle repair after damage and offers a number of advantages. In particular, cryoinjury can be performed such that the size of the lesion and severity of damage are highly similar across experimental animals, with preservation of regenerative muscle satellite cells that nucleate repair in the surrounding uninjured area (Gayraud-Morel, 2009; Dumont et al, 2015; Hardy et al, 2016).

### Histology and CSA quantification

For cryosections, harvested TA muscles were flash-frozen in 2-methylbutane for 30 s followed by liquid nitrogen for 30 s. Samples were stored at −80°C before sectioning. Mouse hearts were rinsed with 1× PBS, embedded in compound at optimal cutting temperature, and frozen. TA samples and cardiomyocyte samples were sectioned at 10 *µ*m. Hematoxylin and eosin (H&E) staining was used to visualize cardiomyocyte cross sections and regenerating myofibers in injured muscles. Sections were stained with hematoxylin for 3 min and washed with tap water for 2 min, followed by back-to-back washes in acid alcohol. Scott’s Bluing Reagent was used for 3 min for nuclear staining, followed by another tap water wash, and finally 2 min for eosin staining. Afterward, sections were dehydrated in ethanol and xylene, and subsequently mounted in Permount Mounting Medium (Cat. #17986-01; Electron Microscopy Sciences) and cured for 24 h before imaging. In regenerating myofibers after cryoinjury, a cross section representing the mid-belly of the TA where there was a clear representation of the injury was chosen. To quantify muscle fiber size, a series of images were taken spanning the entire regenerating area in the cross sections using a dual-head Olympus B×4 microscope with cellSens Standard software. Centrally nucleated myofibers were measured in each image using FIJI software (scale bar = 100 *µ*m), resulting in ∼300–1,500 fibers collectively for each animal. Comparative analyses of more than two groups were performed by one-way ANOVA with Tukey’s correction for multiple comparisons. All analyses were performed under conditions in which the analyst was blinded to sample identity.

### Statistical analyses

All data are presented as mean + SEM. For all data, *n* equals the number of biological replicates of animals used per experiment. The number of animals used for each group was determined based on total empirical data and anticipated completeness of datasets and was sufficient to detect differences in experimental outcomes, if present. Statistical analysis was performed using Prism 8.4.2 for macOS. Comparisons between two different experimental groups were assessed for statistical significance using a *t* test, and comparisons between more than two groups were performed by one-way ANOVA with Tukey’s correction for multiple comparisons. Statistical significance was accepted at *P* < 0.05. All experiments and analyses were performed under blinded conditions.

## Supplementary Material

Reviewer comments
