# Supplementary material for: Functional substitutions of amino acids that differ between GDF11 and GDF8 impact skeletal development and skeletal muscle
Source: Life Sci Alliance. 2023 Jan 11;6(3):e202201662. doi: 10.26508/lsa.202201662 (PMC9834663; doi:10.26508/lsa.202201662)
Supplement: Supplementary file 1 [file LSA-2022-01662_TableS1.docx]

| Mouse Line | *Gdf11^Gdf8aa^* | | | *Gdf8^Gdf11aa^* | | | *Gdf8^Gdf11MD^* | | |
| --- | --- | --- | --- | --- | --- | --- | --- | --- | --- |
| **Genotype** | **+/+** | **+/8aa** | **8aa/8aa** | **+/+** | **+/11aa** | **11aa/11aa** | **+/+** | **+/11MD** | **11MD/11MD** |
|  |  |  |  |  |  |  |  |  |  |
| **F4 Breeding Pairs**  **(Round 1)** | **F5 Progeny**  **(Round 1)** | | | **F5 Progeny**  **(Round 1)** | | | **F5 Progeny**  **(Round 1)** | | |
| +/X x +/X | 2 (20.0) | 5 (50.0) | 3 (30.0) | 3 (30.0) | 4 (40.0) | 3 (30.0) | 4 (25.0) | 8 (50.0) | 4 (25.0) |
| +/X x X/X | -- | 5 (55.6) | 4 (44.4) | -- | 4 (44.4) | 5 (55.6) | -- | 5 (55.6) | 4 (44.4) |
| X/X x X/X | -- | -- | 8 (100) | -- | -- | 10 (100) | -- | -- | 7 (100) |
| **(Round 2)** | **(Round 2)** | | | **(Round 2)** | | | **(Round 2)** | | |
| +/X x +/X | 3 (23.1) | 6 (46.2) | 4 (30.7) | 2 (18.2) | 5 (45.5) | 4 (36.3) | 3 (25.0) | 5 (41.7) | 4 (33.3) |
| +/X x X/X | -- | 5 (62.5) | 3 (37.5) | -- | 4 (50.0) | 4 (50.0) | -- | 4 (50.0) | 4 (50.0) |
| X/X x X/X | -- | -- | 9 (100) | -- | -- | 11 (100) | -- | -- | 11 (100) |
| **(Round 3)** | **(Round 3)** | | | **(Round 3)** | | | **(Round 3)** | | |
| +/X x +/X | 3 (30.0) | 5 (50.0) | 2 (20.0) | 3 (30.0) | 5 (50.0) | 2 (20.0) | 4 (21.1) | 10 (52.6) | 5 (26.3) |
| +/X x X/X | -- | 4 (40.0) | 6 (60.0) | -- | 6 (54.5) | 5 (45.5) | -- | 7 (58.3.0) | 5 (41.7) |
| X/X x X/X | -- | -- | 10 (100) | -- | -- | 9 (100) | -- | -- | 6 (100) |
| **(Total)** | **(Total)** | | | **(Total)** | | |  | **(Total)** |  |
| +/X x +/X | 8 (24.2) | 16 (48.5) | 9 (27.3) | 8 (25.8) | 14 (45.2) | 9 (29.0) | 11 (24.2) | 23 (48.5) | 13 (27.3) |
| +/X x X/X | -- | 14 (51.9) | 13 (48.1) | -- | 14 (50.0) | 14 (50.0) | -- | 16 (55.2) | 13 (44.8) |
| X/X x X/X | -- | -- | 27 (100) | -- | -- | 30 (100) | -- | -- | 24 (100) |
| ** Percentage of genotypes shown in parentheses ( ).* | | | |  |  |  |  |  |  |

**Supplemental Table 1. Genotype and survival distribution of *Gdf11^Gdf8aa^*, *Gdf8^Gdf11aa^*, and *Gdf8^Gdf11MD^* F5 progeny in C57BL/6J background through three initial rounds of breeding.** Progeny of *Gdf11^Gdf8aa^*, *Gdf8^Gdf11aa^*, and *Gdf8^Gdf11MD^* crosses were genotyped and analyzed to confirm Mendelian ratios of F5 progeny and rule out potential lethality as a result of the designed genetic modifications.
