# Supplementary material for: Functional substitutions of amino acids that differ between GDF11 and GDF8 impact skeletal development and skeletal muscle
Source: Life Sci Alliance. 2023 Jan 11;6(3):e202201662. doi: 10.26508/lsa.202201662 (PMC9834663; doi:10.26508/lsa.202201662)
Supplement: Reviewer comments [file LSA-2022-01662_review_history.pdf]

Life Science Alliance

Functional substitutions of amino acids between GDF11 & GDF8 impact skeletal development and muscle.

John Lian, Ryan Walker, Andrea D'Amico, Ana Vujic, Melanie Mills, Kathleen Messemer, Kourtney Mendello, Jill Goldstein, Krystynne Leacock, Soraya Epp, Emma Stimpfl, Thomas Thompson, Amy Wagers, and Richard Lee
DOI: https://doi.org/10.26508/lsa.202201662

Corresponding author(s): Richard Lee, Harvard University and John Lian, Harvard University

Review Timeline:

Submission Date:	2022-08-09
Editorial Decision:	2022-09-12
Revision Received:	2022-12-07
Editorial Decision:	2022-12-08
Revision Received:	2022-12-11
Accepted:	2022-12-12

Scientific Editor: Eric Sawey, PhD

Transaction Report:

(Note: With the exception of the correction of typographical or spelling errors that could be a source of ambiguity, letters and reports are not edited. The original formatting of letters and referee reports may not be reflected in this compilation.)

September 12, 2022

Re: Life Science Alliance manuscript #LSA-2022-01662-T

Dr. Richard T Lee
Harvard University
Harvard Stem Cell Institute and Department of Stem Cell and Regenerative Biology
Department of Medicine Harvard Medical School Brigham and Women's Hospita 75 Francis St.
Boston, MA 02115

Dear Dr. Lee,

Thank you for submitting your manuscript entitled "Functional substitutions of amino acids that differ between GDF11 and GDF8 impact skeletal development and muscle." to Life Science Alliance. The manuscript was assessed by expert reviewers, whose comments are appended to this letter. We invite you to submit a revised manuscript addressing the Reviewer comments.

To upload the revised version of your manuscript, please log in to your account: https://lsa.msubmit.net/cgi-bin/main.plex

You will be guided to complete the submission of your revised manuscript and to fill in all necessary information. Please get in touch in case you do not know or remember your login name.

While you are revising your manuscript, please also attend to the below editorial points to help expedite the publication of your manuscript. Please direct any editorial questions to the journal office.

The typical timeframe for revisions is three months. Please note that papers are generally considered through only one revision cycle, so strong support from the referees on the revised version is needed for acceptance.

When submitting the revision, please include a letter addressing the reviewers' comments point by point.

We hope that the comments below will prove constructive as your work progresses.

Thank you for this interesting contribution to Life Science Alliance. We are looking forward to receiving your revised manuscript.

Sincerely,

Eric Sawey, PhD
Executive Editor
Life Science Alliance
http://www.lsajournal.org

A. THESE ITEMS ARE REQUIRED FOR REVISIONS

-- A letter addressing the reviewers' comments point by point.

-- An editable version of the final text (.DOC or .DOCX) is needed for copyediting (no PDFs).

-- High-resolution figure, supplementary figure and video files uploaded as individual files: See our detailed guidelines for preparing your production-ready images, https://www.life-science-alliance.org/authors

-- Summary blurb (enter in submission system): A short text summarizing in a single sentence the study (max. 200 characters including spaces). This text is used in conjunction with the titles of papers, hence should be informative and complementary to the title and running title. It should describe the context and significance of the findings for a general readership; it should be written in the present tense and refer to the work in the third person. Author names should not be mentioned.

-- By submitting a revision, you attest that you are aware of our payment policies found here: https://www.life-science-alliance.org/copyright-license-fee

B. MANUSCRIPT ORGANIZATION AND FORMATTING:

Full guidelines are available on our Instructions for Authors page, https://www.life-science-alliance.org/authors

We encourage our authors to provide original source data, particularly uncropped/-processed electrophoretic blots and spreadsheets for the main figures of the manuscript. If you would like to add source data, we would welcome one PDF/Excel-file per figure for this information. These files will be linked online as supplementary "Source Data" files.

IMPORTANT: It is Life Science Alliance policy that if requested, original data images must be made available. Failure to provide original images upon request will result in unavoidable delays in publication. Please ensure that you have access to all original microscopy and blot data images before submitting your revision.

Reviewer #1 (Comments to the Authors (Required)):

This was a very rigorous paper that dove deeply into the differences between GDF8 versus GDF11, and more specifically at the level type I receptor binding interface and the amino acids that differ between these proteins. The results are interesting and show alterations in axial skeletal development suggesting how these 2 regions differ in vivo in how each protein functions. Overall the study is very well done and it represents a great deal of work. The study is also very straightforward and I do not have any significant technical concerns or problems with data presentation or interpretation. My only comment is that the work is highly focused and reductionary, and it might only be of interest to experts in the field. Despite this concern, the study is meritorious and describes mice and a resulting functional phenotype that better distinguishes GDF8 from GDF11, again supporting the concept that these 2 related family members do not overlap in function

Reviewer #2 (Comments to the Authors (Required)):

Lian and Walker et al. Work focuses in two highly homologous TGFB proteins, GDF11 and GDF8 (also known as myostatin) and their role and biological actions in musculoskeletal development and muscle regeneration. Remarkably, the authors challenge previous -and recent findings- about the role of GDF11 and GFD8 activities in vivo using CRISPR/CAS transgenic and chimeric mice in which they substituted key structural amino acids (signalling domains) that differ between these two GDF proteins. Here, the authors show differential in in vivo and unique roles of GDF11 and 8, which cannot be merely explained by differential ligand concentration and patterns of expression. First, they show decrease GDF11 potency when replacing these key amino acids with those of GDF8, and therefore affected function, cause defects in axial musculoskeletal patterning, craniofacial bone, and limb development, in which in some the defects seem to recapitulate previous findings using KO models. However, no over phenotype was found in mice lacking GDF8, except for minor effects in muscle mass (atrophy? Development?). Overall, this study reveals distinct roles of GDF11 and 8 mature domains in vivo based on two key signalling domain-associated amino acids. Note, I am pleased to see the authors put their work in context with a recent (and quite similar) manuscript just published, well done.

Comments and revisions:
1. Please clarify what potency refers in the introduction, paragraph 4.
2. The authors indicate that previous studies have shown that administration of rGDF11 to aged mice reduces cardiac hypetrophy, but then they evaluated some heart parameters (weight, cardiomyocyte CSA, and others) in young mice using their transgenic lines. Why is that? I think there is some miscommunication to what has been demonstrated and their goals in evaluating a cardiac function of GDF11 or GDF8. Please, clarify or explain better the argumentative rationale behind.
3. How could the serum concentrations correlate with the tissue concentration? I ask this because I assume that the extracellular binding capabilities of both molecules are not affected, or are they?
4. Figure 3. Could the authors show pictures from above the cranium?
5. Figures 4, 5 and 6 are hard to read, and therefore, interpret. Please, increase the font size for enhancing eligibility.
6. Table 2. Please add the age at which these parameters were evaluated. I understand this info can be found in M&M section, but it would be helpful to have it here too.
7. At what time during development do the authors see a developmental failure of muscle formation in the Gdf8Gdf11MD? Are there any defects in limb muscle patterning? In that line, what are the cell type(s) responsible for it? What about other muscle groups like craniofascial muscles or diaphragm?

Reviewer #1:

"This was a very rigorous paper that dove deeply into the differences between GDF8 versus GDF11, and more specifically at the level type I receptor binding interface and the amino acids that differ between these proteins. The results are interesting and show alterations in axial skeletal development suggesting how these 2 regions differ in vivo in how each protein functions. Overall, the study is very well done, and it represents a great deal of work. The study is also very straightforward, and I do not have any significant technical concerns or problems with data presentation or interpretation. My only comment is that the work is highly focused and reductionary, and it might only be of interest to experts in the field. Despite this concern, the study is meritorious and describes mice and a resulting functional phenotype that better distinguishes GDF8 from GDF11, again supporting the concept that these 2 related family members do not overlap in function."

Author Response: Thank you for your positive feedback. We understand that our study is highly focused, but we suggest that there is broad interest in these ligands. It is particularly significant that many recent clinical trials on inhibiting GDF8 function have failed in patients. Therefore, we believe that the molecular differences between the molecules *in vivo* are an important topic, and our study represents a step forward worthy of publication.

Reviewer #2:

"Lian and Walker et al. Work focuses in two highly homologous TGFB proteins, GDF11 and GDF8 (also known as myostatin) and their role and biological actions in musculoskeletal development and muscle regeneration. Remarkably, the authors challenge previous -and recent findings- about the role of GDF11 and GFD8 activities in vivo using CRISPR/CAS transgenic and chimeric mice in which they substituted key structural amino acids (signaling domains) that differ between these two GDF proteins. Here, the authors show differential in in vivo and unique roles of GDF11 and 8, which cannot be merely explained by differential ligand concentration and patterns of expression. First, they show decrease GDF11 potency when replacing these key amino acids with those of GDF8, and therefore affected function, cause defects in axial musculoskeletal patterning, craniofacial bone, and limb development, in which in some the defects seem to recapitulate previous findings using KO models. However, no over phenotype was found in mice lacking GDF8, except for minor effects in muscle mass (atrophy? Development?). Overall, this study reveals distinct roles of GDF11 and 8 mature domains in vivo based on two key signaling domain-associated amino acids. Note, I am pleased to see the authors put their work in context with a recent (and quite similar) manuscript just published, well done."

"Comments and revisions:

1. Please clarify what potency refers in the introduction, paragraph 4."

Author Response: Thank you for your helpful advice and detailed suggestions to help make our manuscript easier to comprehend. We have added a definition for "potency" as it relates to our study and clarified in paragraph 4 of the Introduction that "potency" refers to "signaling potency" differences between GDF11 and GDF8 ligands with amino acid substitutions, previously shown in Walker et al., 2017.

Revised manuscript:

Introduction, Paragraph 3.

> "In a prior study, we demonstrated that GDF11 and GDF8 differ in their signaling properties in multiple cell lines and cultured primary myoblasts, with GDF11 signaling at lower concentrations than GDF8 and more efficiently utilizing the type I receptors ALK4, ALK5, and ALK7 (Walker et al., 2017). We define this ability to activate downstream pathways at lower concentrations as having greater *potency*."

Introduction, Paragraph 4.

> "These sequence alterations in GDF11, which previously were shown to diminish signaling potency of the resulting protein (Walker et al., 2017), caused a perturbation of the axial skeletal structure of mutant mice during development that persists into adulthood. In contrast, the sequence alterations introduced into GDF8, which previously were shown to increase the signaling potency of the resulting ligand (Walker et al., 2017), did not produce observable developmental phenotypes."

“2. The authors indicate that previous studies have shown that administration of rGDF11 to aged mice reduces cardiac hypertrophy, but then they evaluated some heart parameters (weight, cardiomyocyte CSA, and others) in young mice using their transgenic lines. Why is that? I think there is some miscommunication to what has been demonstrated and their goals in evaluating a cardiac function of GDF11 or GDF8. Please, clarify or explain better the argumentative rationale behind.”

Author Response: Thank you for the suggestion. With dose response experiments that accounted for differences in protein quality, exogenous recombinant GDF11 does reduce cardiac hypertrophy in old mice as well as young mice (Pogglioli et al, *Circ Res* 2015 & Harper et al, *Circ Res* 2018). Because *in vitro* experiments show that GDF11 is a more potent ligand than GDF8, there was potential for the genetically engineered mice to have changes in cardiac parameters. We agree that aging the mice will be an interesting analysis, and we have clarified our rationale for characterizing heart parameters, as follows:

Revised manuscript:

Introduction, Paragraph 2.

> “Importantly, *Gdf11*-null mice exhibit perinatal lethality, whereas *Mstn*-null (*Gdf8*$^{-/-}$) mice do not (McPherron et al., 1999), and lower levels of *Mstn* in *Gdf8*$^{+/-}$ heterozygotes may actually extend lifespan (Mendias et al., 2015). These differences in postnatal survival following genetic manipulation make comparative studies of *Gdf11* versus *Gdf8* activities *in vivo* particularly difficult, while reports on GDF11’s essential functions in adulthood—most of which have relied on the use of exogenous recombinant proteins—are incompletely defined.”

Results, *Baseline cardiac physiology and function remains unchanged in Gdf11*^{Gdf8aa}*, Gdf8*^{Gdf11aa}*, and Gdf8*^{Gdf11MD} *mutants*, Paragraph 1.

> “Previous studies have shown that administration of exogenous recombinant GDF11 to aged mice reduces cardiac hypertrophy (Loffredof et al., 2013), and fetal cardiac GDF8 has also been implicated in early-stage heart development (Sharma et al., 1999). Since our *in vitro* experiments showed that GDF11 is a more potent signaling ligand than GDF8, there is potential for the genetically engineered mutants to have changes in cardiac parameters.”

“3. How could the serum concentrations correlate with the tissue concentration? I ask this because I assume that the extracellular binding capabilities of both molecules are not affected, or are they?”

Author Response: We have not measured tissue concentrations, and the mass spectrometry assay has been validated only for serum. Based on the biochemistry of the ligands, we do not anticipate that the matrix binding of the ligands would be affected, but we have not studied this rigorously. We have modified the manuscript as follows:

Revised manuscript:

Results, *Circulating GDF11 concentration increases 50-fold in Gdf8^{Gdf11MD} mutants, while GDF11 and GDF8 levels in Gdf11^{Gdf8aa} and Gdf8^{Gdf11aa} mutants remain unchanged*, Paragraph 1.

> “Our study further expands this analysis of the impact of mature domain sequence on ligand expression by measuring endogenous GDF11 and GDF8 in serum from *Gdf11*^{Gdf8aa} and *Gdf8*^{Gdf11aa} mutants as well, showing that serum GDF11 and GDF8 protein concentrations are not significantly altered in either mono-allelic or bi-allelic *Gdf11*^{Gdf8aa} or *Gdf8*^{Gdf11aa} mutants, compared to wild-type mice **(Fig. 2A, B, C).** Based on the biochemistry of the ligands, we do not anticipate that the matrix binding of GDF11 and GDF8 would be affected, though we have not studied this.”

“4. Figure 3. Could the authors show pictures from above the cranium?”

Author Response: We have added to Figure 3 an additional panel of images showing the cranium from above.

Revised manuscript:

Figure 3.

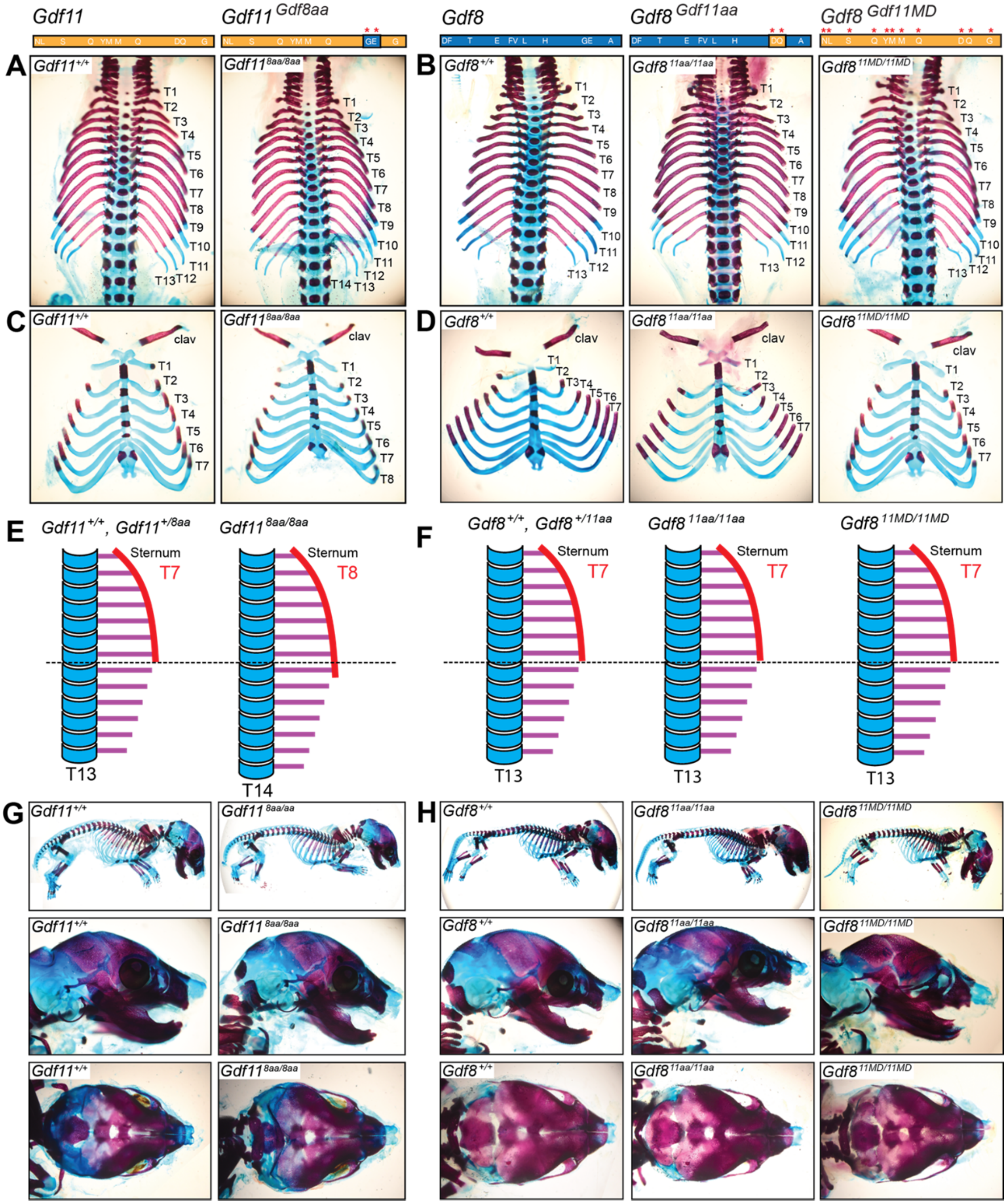

“5. Figures 4, 5 and 6 are hard to read, and therefore, interpret. Please, increase the font size for enhancing legibility.”

Author Response: Thank you for this suggestion. In Figures 4, 5, and 6, we have enlarged the overall font size, consolidated the titles of each muscle group, and updated the organization of these figures to make them easier to read. For consistency, we have made the same formatting changes to Figure 2 (and all supplemental figures), as follows:

Revised manuscript:

Figure 2.

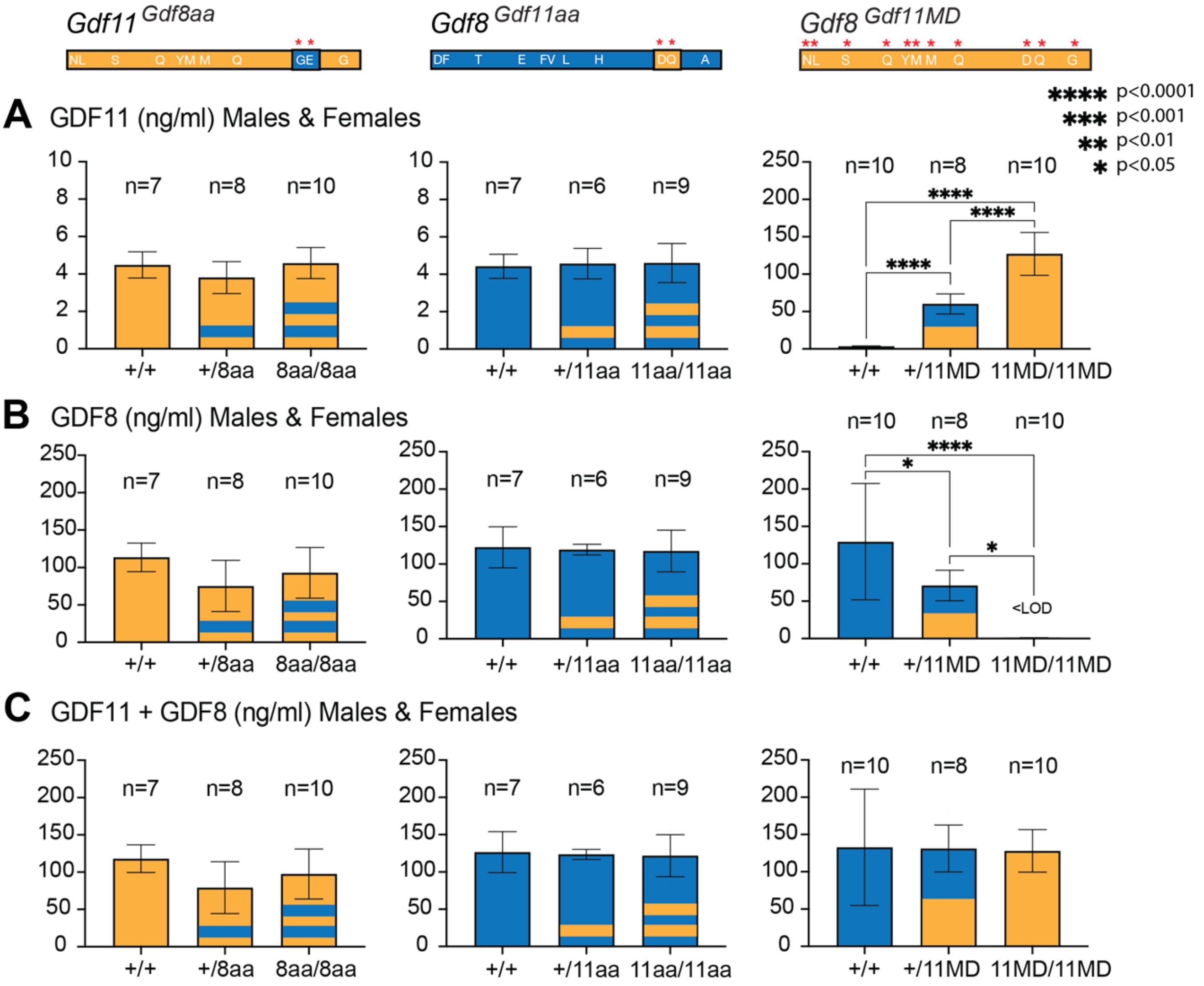

Figure 4.

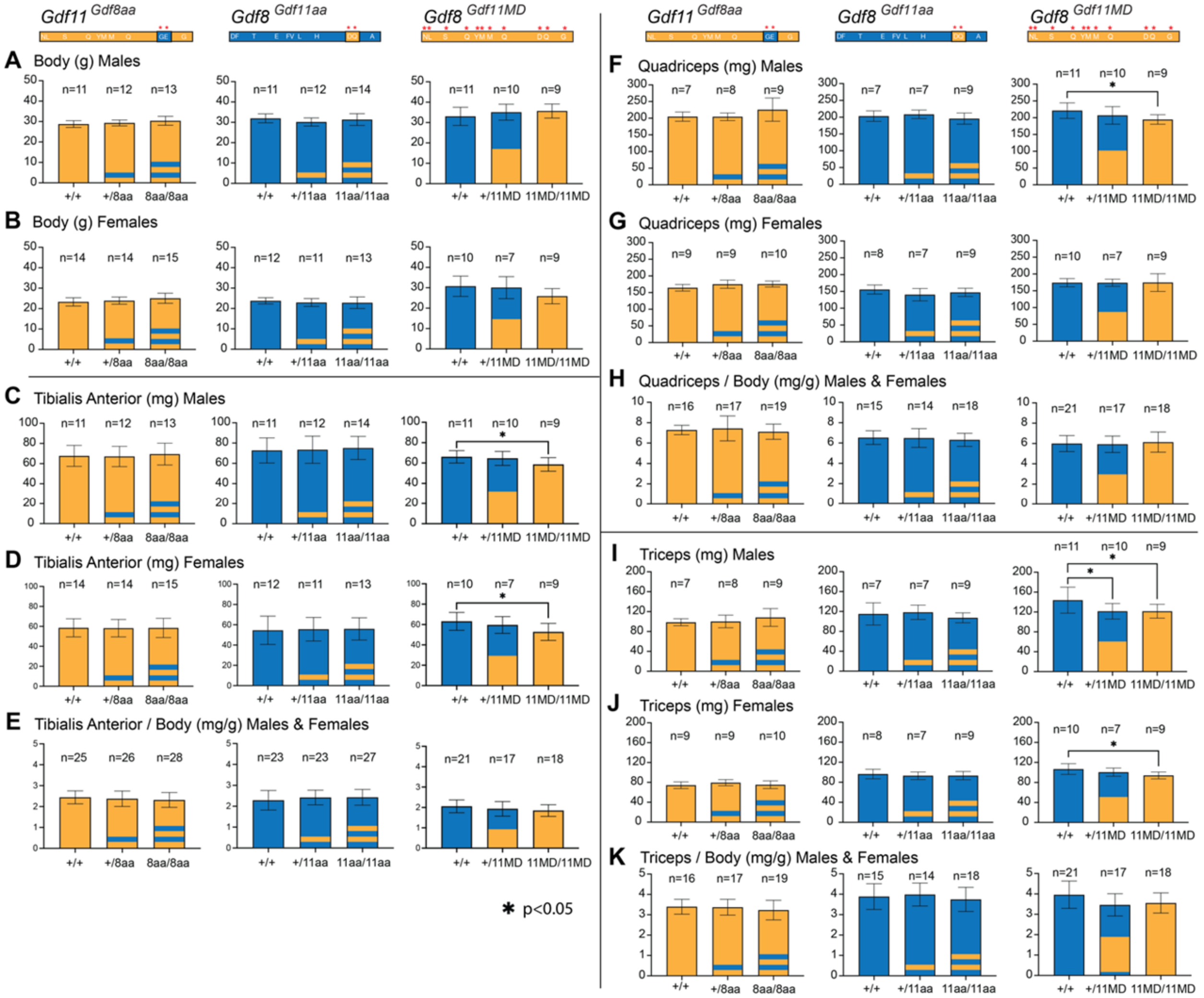

Figure 5.

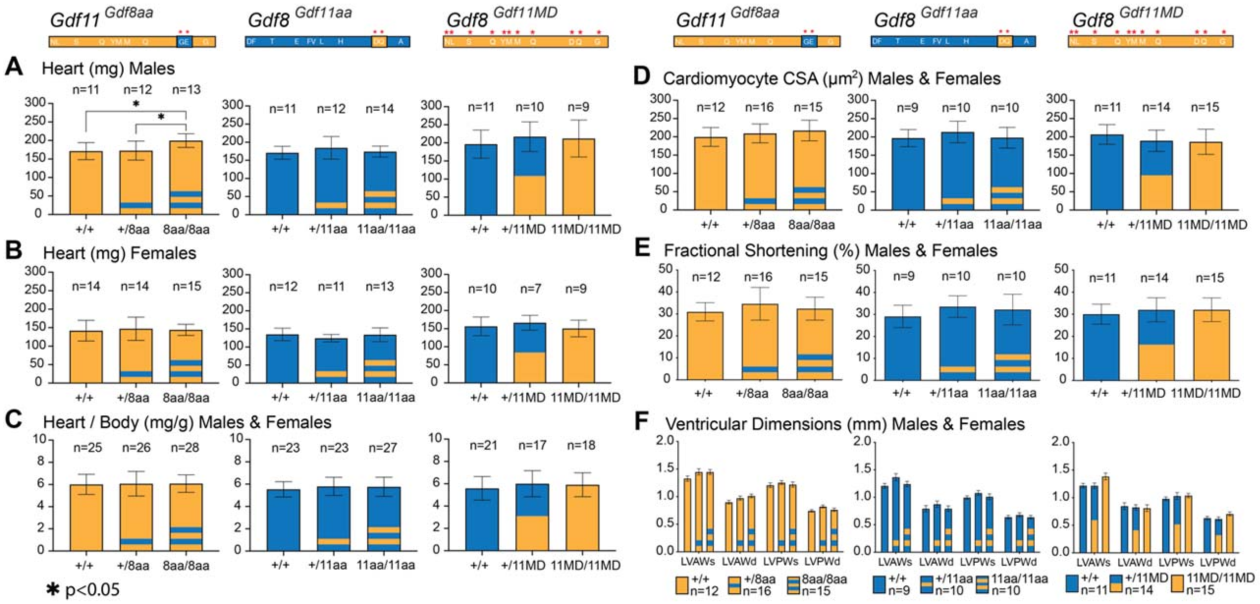

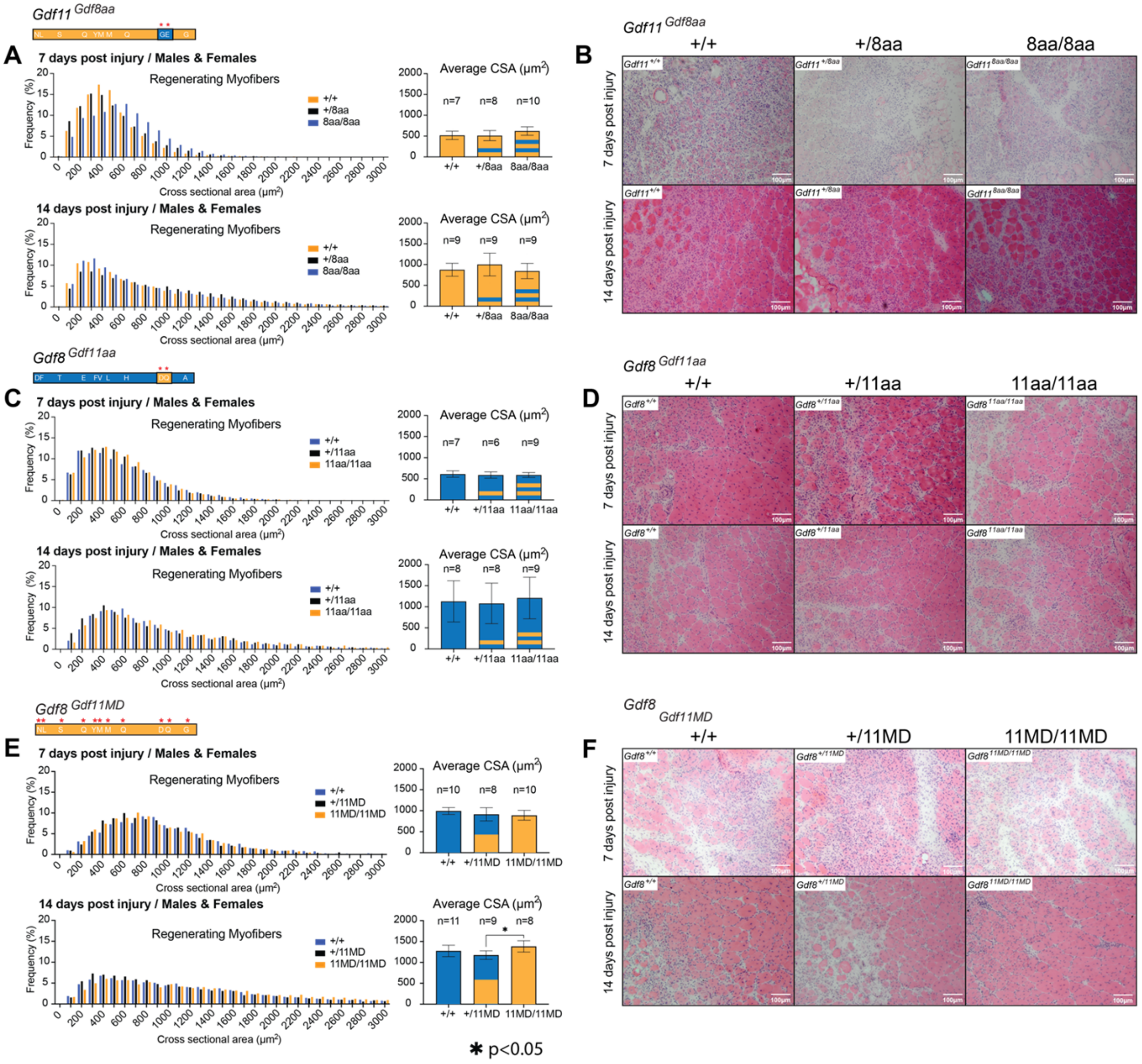

Gdf11 Gdf8aa
A
7 days post injury / Males & Females
Regenerating Myofibers
+/+
+/8aa
8aa/8aa
Frequency (%)
Cross sectional area (µm²)
Average CSA (µm²)
n=7
n=8
n=10
14 days post injury / Males & Females
n=9
n=9
n=9
B
7 days post injury
14 days post injury
Gdf8 Gdf11aa
C
+/11aa
11aa/11aa
n=7
n=6
n=9
n=8
n=8
n=9
D
Gdf8 Gdf11MD
E
+/11MD
11MD/11MD
n=10
n=8
n=10
n=11
n=9
n=8
F
✱ p<0.05

Figure 6.

“6. Table 2. Please add the age at which these parameters were evaluated. I understand this info can be found in M&M section, but it would be helpful to have it here too.”

Author Response: Thank you for the suggestion. We have added to Table 2 the mice ages and timepoints at which each parameter was measured, as below:

Revised manuscript:

Table 2.

**Tissue / Phenotype**	**GDF11**	**GDF8**	
Predominant Expression Pattern	Developing limb buds; Primitive streak and tail bud	Development and adult skeletal muscle maintenance	
**Chimeric Lines**	***Gdf11^{Gdf8aa}***	***Gdf8^{Gdf11aa}***	***Gdf8^{Gdf11MD}***
Premature lethality	No	No	No
Bone (E18.5)	**Transformation of the axial skeleton (T14 thoracic vertebrae, T8 vertebrosternal ribs)**	No difference compared to wild-type	No difference compared to wild-type
Circulating ligand concentration (10-14 wks)	No difference compared to wild-type	No difference compared to wild-type	**~50-fold increase in GDF11 in bi-allelic mutants; GDF8 levels at or below level of detection**
Adult skeletal muscle (10-14 wks)	No difference compared to wild-type	No difference compared to wild-type	**Statistically significant decrease in mutant tibialis anterior, quadriceps, and triceps weights**
Heart (10-14 wks)	**Statistically significant increase in mutant male heart weights; not observed in mutant females**	No difference in function and physiology compared to wild-type	No difference in function and physiology compared to wild-type
Cardiac myocytes (10-14 wks)	No difference in cross-sectional area compared to wild-type	No difference in cross-sectional area compared to wild-type	No difference in cross-sectional area compared to wild-type
Kidney (10-14 wks)	Normal compared to wild-type; no observed renal agenesis	Normal compared to wild-type; no observed renal agenesis	Normal compared to wild-type; no observed renal agenesis
Liver (10-14 wks)	Observed slight increase in bi-allelic mutant liver weight, however not statistically significant	No difference compared to wild-type	**Statistically significant decrease in bi-allelic mutant female liver weight; not observed in mutant males**
Injured muscle regeneration (10-14 wks, harvest at 7d & 14d post-injury)	No difference compared to wild-type	No difference compared to wild-type	No difference compared to wild-type

"7. At what time during development do the authors see a developmental failure of muscle formation in the Gdf8Gdf11MD? Are there any defects in limb muscle patterning? In that line, what are the cell type(s) responsible for it? What about other muscle groups like craniofacial muscles or diaphragm?"

Author Response: We did not observe a specific timepoint during development at which muscle formation abruptly stops or fails, rather the development of the chimeric mice appeared gradual and normal. There were no distinct measurable defects or patterning malformations in other muscle groups, such as craniofacial muscles or diaphragm, either. For measuring muscle regeneration capacity of the mutant mice, we damaged the tibialis anterior (TA) muscle via cryoinjury in order to activate quiescent satellite cells in the basal lamina of myofibers, then harvested the muscle at 7 and 14 days post-injury. However, we observed normal muscle regeneration capability similar to wild-type. We have modified the manuscript as below:

Revised manuscript:

Results, *$Gdf8^{Gdf11MD}$ mutants exhibit decreased skeletal muscle mass, while the muscles of $Gdf11^{Gdf8aa}$ and $Gdf8^{Gdf11aa}$ mutants are not significantly altered*, Paragraph 2.

> "Overall, we did not observe any distinct defects or malformations in muscle development or patterning at any point during development, and the chimeric mice appeared similar to wild-type into early adulthood. In all cases, separation by males and females resulted in shifts in the mean muscle mass between the sexes."

December 8, 2022

RE: Life Science Alliance Manuscript #LSA-2022-01662-TR

Dr. Richard T Lee
Harvard University
Harvard Stem Cell Institute and Department of Stem Cell and Regenerative Biology
Department of Medicine Harvard Medical School Brigham and Women's Hospita 75 Francis St.
Boston, MA 02115

Dear Dr. Lee,

Thank you for submitting your revised manuscript entitled "Functional substitutions of amino acids between GDF11 & GDF8 impact skeletal development and muscle.". We would be happy to publish your paper in Life Science Alliance pending final revisions necessary to meet our formatting guidelines.

Along with points mentioned below, please tend to the following:
-please add ORCID ID for secondary corresponding author-they should have received instructions on how to do so
-please add the Twitter handle of your host institute/organization as well as your own or/and one of the authors in our system
-please add a figure legend section to your main manuscript, including your main and supplementary figures and your table legends
-please upload your table files as editable doc or excel files, or make sure they're included in the doc file of your main manuscript
-we encourage you to introduce the panels in your figure legends in alphabetical order
-please add a callout for Figure S3A and Figure S1D, E, F, I to your main manuscript text
-please mention panel H in the Figure 3 legend

If you are planning a press release on your work, please inform us immediately to allow informing our production team and scheduling a release date.

LSA now encourages authors to provide a 30-60 second video where the study is briefly explained. We will use these videos on social media to promote the published paper and the presenting author (for examples, see https://twitter.com/LSAjournal/timelines/1437405065917124608). Corresponding or first-authors are welcome to submit the video. Please submit only one video per manuscript. The video can be emailed to contact@life-science-alliance.org

To upload the final version of your manuscript, please log in to your account: https://lsa.msubmit.net/cgi-bin/main.plex
You will be guided to complete the submission of your revised manuscript and to fill in all necessary information. Please get in touch in case you do not know or remember your login name.

To avoid unnecessary delays in the acceptance and publication of your paper, please read the following information carefully.

A. FINAL FILES:

These items are required for acceptance.

-- An editable version of the final text (.DOC or .DOCX) is needed for copyediting (no PDFs).

-- High-resolution figure, supplementary figure and video files uploaded as individual files: See our detailed guidelines for preparing your production-ready images, https://www.life-science-alliance.org/authors

-- Summary blurb (enter in submission system): A short text summarizing in a single sentence the study (max. 200 characters including spaces). This text is used in conjunction with the titles of papers, hence should be informative and complementary to the title. It should describe the context and significance of the findings for a general readership; it should be written in the present tense and refer to the work in the third person. Author names should not be mentioned.

B. MANUSCRIPT ORGANIZATION AND FORMATTING:

Full guidelines are available on our Instructions for Authors page, https://www.life-science-alliance.org/authors

We encourage our authors to provide original source data, particularly uncropped/-processed electrophoretic blots and spreadsheets for the main figures of the manuscript. If you would like to add source data, we would welcome one PDF/Excel-file

per figure for this information. These files will be linked online as supplementary "Source Data" files.

Submission of a paper that does not conform to Life Science Alliance guidelines will delay the acceptance of your manuscript.

It is Life Science Alliance policy that if requested, original data images must be made available to the editors. Failure to provide original images upon request will result in unavoidable delays in publication. Please ensure that you have access to all original data images prior to final submission.

The license to publish form must be signed before your manuscript can be sent to production. A link to the electronic license to publish form will be sent to the corresponding author only. Please take a moment to check your funder requirements.

Reviews, decision letters, and point-by-point responses associated with peer-review at Life Science Alliance will be published online, alongside the manuscript. If you do want to opt out of having the reviewer reports and your point-by-point responses displayed, please let us know immediately.

Thank you for your attention to these final processing requirements. Please revise and format the manuscript and upload materials within 7 days.

Thank you for this interesting contribution, we look forward to publishing your paper in Life Science Alliance.

Sincerely,

Eric Sawey, PhD
Executive Editor
Life Science Alliance
http://www.lsajournal.org

December 12, 2022

RE: Life Science Alliance Manuscript #LSA-2022-01662-TRR

Dr. Richard T Lee
Harvard University
Harvard Stem Cell Institute and Department of Stem Cell and Regenerative Biology
Department of Medicine Harvard Medical School Brigham and Women's Hospita 75 Francis St.
Boston, MA 02115

Dear Dr. Lee,

Thank you for submitting your Research Article entitled "Functional substitutions of amino acids between GDF11 & GDF8 impact skeletal development and muscle". It is a pleasure to let you know that your manuscript is now accepted for publication in Life Science Alliance. Congratulations on this interesting work.

The final published version of your manuscript will be deposited by us to PubMed Central upon online publication.

Your manuscript will now progress through copyediting and proofing. It is journal policy that authors provide original data upon request.

Reviews, decision letters, and point-by-point responses associated with peer-review at Life Science Alliance will be published online, alongside the manuscript. If you do want to opt out of having the reviewer reports and your point-by-point responses displayed, please let us know immediately.

IMPORTANT: If you will be unreachable at any time, please provide us with the email address of an alternate author. Failure to respond to routine queries may lead to unavoidable delays in publication.

Scheduling details will be available from our production department. You will receive proofs shortly before the publication date. Only essential corrections can be made at the proof stage so if there are any minor final changes you wish to make to the manuscript, please let the journal office know now.

DISTRIBUTION OF MATERIALS:
Authors are required to distribute freely any materials used in experiments published in Life Science Alliance. Authors are encouraged to deposit materials used in their studies to the appropriate repositories for distribution to researchers.

You can contact the journal office with any questions, contact@life-science-alliance.org

Again, congratulations on a very nice paper. I hope you found the review process to be constructive and are pleased with how the manuscript was handled editorially. We look forward to future exciting submissions from your lab.

Sincerely,

Eric Sawey, PhD
Executive Editor
Life Science Alliance
http://www.lsajournal.org
